# Dual fluorination of polymer electrolyte and conversion-type cathode for high-capacity all-solid-state lithium metal batteries

**DOI:** 10.1038/s41467-022-35636-0

**Published:** 2022-12-23

**Authors:** Jiulin Hu, Chuanzhong Lai, Keyi Chen, Qingping Wu, Yuping Gu, Chenglong Wu, Chilin Li

**Affiliations:** 1 State Key Laboratory of High Performance Ceramics and Superfine Microstructure, Shanghai Institute of Ceramics, Chinese Academy of Sciences, 585 He Shuo Road, 201899 Shanghai, China; 2 CAS Key Laboratory of Materials for Energy Conversion, Shanghai Institute of Ceramics, Chinese Academy of Sciences, 201899 Shanghai, China; 3 Center of Materials Science and Optoelectronics Engineering, University of Chinese Academy of Sciences, 100049 Beijing, China

**Keywords:** Batteries, Energy science and technology, Materials for energy and catalysis, Energy, Electrochemistry

## Abstract

All-solid-state batteries are appealing electrochemical energy storage devices because of their high energy content and safety. However, their practical development is hindered by inadequate cycling performances due to poor reaction reversibility, electrolyte thickening and electrode passivation. Here, to circumvent these issues, we propose a fluorination strategy for the positive electrode and solid polymeric electrolyte. We develop thin laminated all-solid-state Li||FeF_3_ lab-scale cells capable of delivering an initial specific discharge capacity of about 600 mAh/g at 700 mA/g and a final capacity of about 200 mAh/g after 900 cycles at 60 °C. We demonstrate that the polymer electrolyte containing AlF_3_ particles enables a Li-ion transference number of 0.67 at 60 °C. The fluorinated polymeric solid electrolyte favours the formation of ionically conductive components in the Li metal electrode’s solid electrolyte interphase, also hindering dendritic growth. Furthermore, the F-rich solid electrolyte facilitates the Li-ion storage reversibility of the FeF_3_-based positive electrode and decreases the interfacial resistances and polarizations at both electrodes.

## Abstract (web summary)

The practical use of all-solid-state batteries is hindered by inadequate cycling performance. Here, the authors propose a fluorination strategy for the positive electrode and polymeric electrolyte to develop all-solid-state Li||FeF3 pouch cells with high discharge capacity and long cycle life.

## Introduction

Pursuing the batteries with enhanced energy density and high safety is of great significance to the development and practical application of electrochemical energy storage devices^1^. Solid-state batteries (SSBs) are constructed by replacing flammable nonaqueous liquid electrolyte solution with thermal stable solid-state electrolytes (SSEs), and they are expected to improve the safety especially when coupled with lithium metal anode^2^. Inorganic SSEs like garnet-type Li_7_La_3_Zr_2_O_12_ (LLZO), NASCON-type Li_1.3_Al_0.3_Ti_1.7_(PO_4_)_3_ (LATP) and sulfide-based Li_10_GeP_2_S_12_ show satisfactory ionic conductivity (usually exceeding 0.1 mS/cm at 30 °C)^3^. However, their large mechanical stiffness and prevailing interface passivation (or side reaction) hinder their practical application^3^. On the other hand, the solid polymer electrolytes (SPEs) have the advantages of scalable manufacture, small interface impedance and optimized operating pressure^4^. Polyethylene oxide (PEO) based polymer electrolytes have been widely studied due to its easy fabrication and flexibility^4^. It is demonstrated that Li-ions can migrate via the vibration of –C–O–C– chain segments and this behavior is affected by the degree of disorder in the PEO microscopic area. However, the Li ion conductivity, mechanical properties and thermal stability are quite poor for pure PEO electrolyte. Adding the functional filler to polymer would improve the comprehensive properties due to the optimization of internal multi-scale interactions and conductive interfaces^5^. Up to now, numerous fillers have been proposed to reinforce the PEO-based SPEs, such as the ionic conductor fillers like LLZO and LATP^6,7^. Although these ion-conductor-type fillers themselves bring sufficient Li-ion conductivity in the composite configurations, the heavy-metal-contained ceramic materials usually require a complex preparation process in order to obtain the tailored nanostructures^8^. Besides, the ceramic/polymer interfaces inside SPEs may also degrade due to the potential side reaction or gravitational separation, significantly affecting the transport performance of SPEs during long-term operation^8^. The porous morphology of fillers is favorable for the increase of crosslinking dimension with PEO chains, like SiO_2_ aerogel and g-C_3_N_4_ holey microspheres^9,10^. In fact, the original intention of most filler strategies is to reinforce the crosslinking with the PEO matrix and at the same time restrain the movement of salt anions (e.g., bis(trifluoromethane)sulfonimide anions, TFSI^−^), thus increasing the Li-ion transference number (*t*_Li+_). However, the in-depth crosslinking mechanism and related charge transport scenario between filler and PEO have not been clearly revealed. The strong interaction between acidic oxide (e.g. Al_2_O_3_) or metal-organic framework (MOF) type filler and PEO-salt is attributed to the surface Lewis acid-base reaction^5,11^. However, the highly effective effort focusing on the adjustable Lewis acid-base mechanism to improve the SPE performance is still lacking.

Fluoride plays a vital role in nonaqueous electrolyte systems in view of its effects on the inhibition of Li anode dendrites and extension of cathodic voltage range^12,13^. The suitable fluorination of solvent molecules can endow both the nonaqueous and aqueous electrolytes with good low-temperature performance^14,15^. The fluoride-based solid electrolytes have also proved to disclose both the satisfactory air stability and ionic conductivity^16,17^. The fluorine doping into traditional oxide and sulfide solid electrolytes can stabilize the interface with electrode and reduce the voltage polarization of corresponding SSBs, such as for the argyrodite and LLZO-based systems^18,19^. As for the fluorinated polymer electrolyte, one typical example is the poly(vinylidene fluoride) (PVDF) or its co-polymer Poly(vinylidene fluoride-co-hexafluoropropylene) (PVDF-HFP), which has extended electrochemical window^20^. However, the dissolution process of PVDF is more difficult than that of PEO, making the molding of the former membrane usually more cumbersome. Besides, the homopolymer structure of PVDF results in a high degree of intramolecular crystallinity (65–78 wt%), which is not ionically conductive^5,21^. Therefore the room temperature (RT) conductivity of PVDF-based electrolyte system is generally low. The interfacial fluorination has been demonstrated to enable the performance improvement of PEO-based electrolyte system. For example, the introduction of Al_2_O_3_ into hygroscopic sodium bis(fluorosulfonyl)imide (NaFSI) dissolved PEO system can realize the in situ formation and aggregation of AlF_3_·*x*H_2_O interface layer, benefitting to the cycle stability and rate performance of batteries^22^. A recent work shows that the F-doped Li_0.33_La_0.557_TiO_3_ (LLTO) can serve as filler of PEO system to reinforce the effect of Li dendrite suppression^23^. The addition of AlF_3_ into the 1,3-dioxolane (DOL) based electrolyte can in situ initiate the ring-opening polymerization of solvent molecules via the Lewis acid-base reaction, leading to the formation of fluorinated solid polymer electrolyte. Its wiring into LiNi_0.6_Co_0.2_Mn_0.2_O_2_ (NCM) cathode enables the achievement of high-voltage Li metal batteries with corrosion mitigation of Al current collector^24^. Although the fluorination treatment has demonstrated some modification effects on electrolyte ionic conductivity, anode and cathode stability, the homogenization and globalization of F content are still a big challenge especially for the PEO system.

**Fig. 1:**
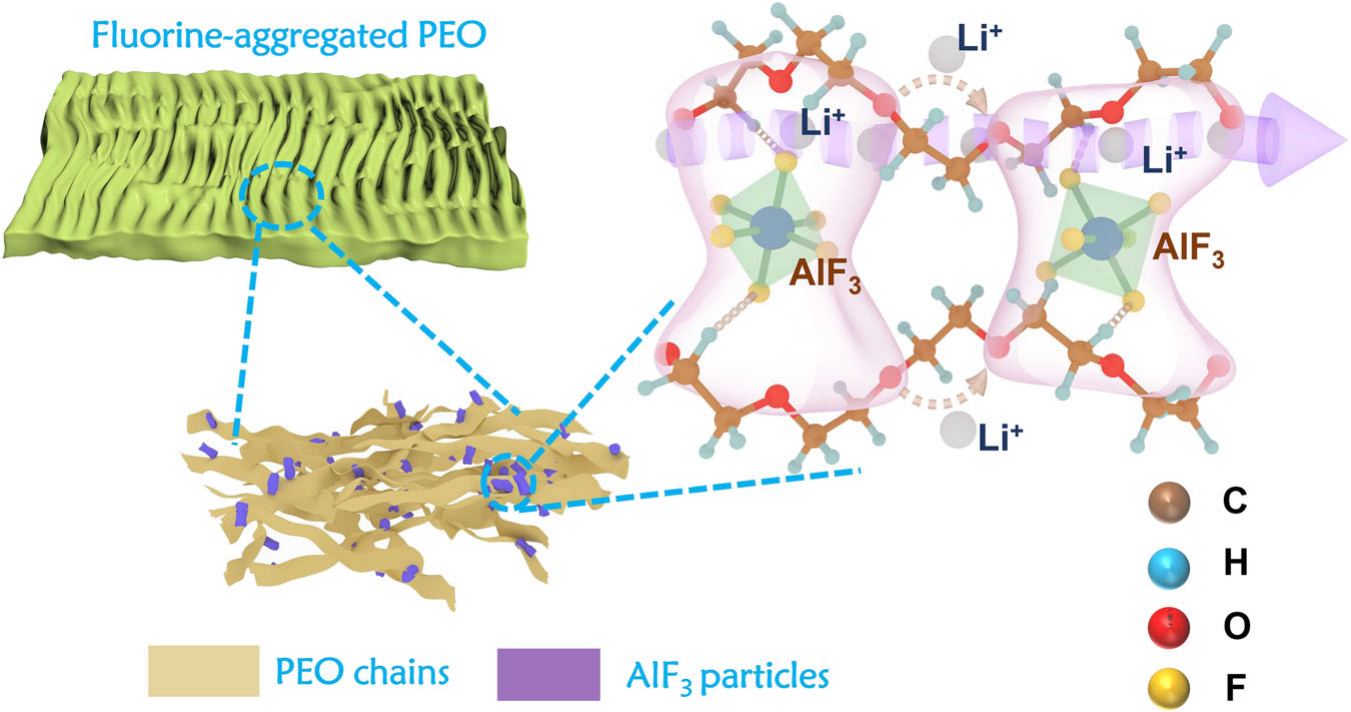
Schematic illustration of fluorinated PEO-based electrolyte. Schemes of electrolyte membrane texture, blending situation of AlF_3_ particles and PEO chains, and “Li-ion transport highway” produced by the strong interaction between HS-AlF_3_ and PEO.

On the other hand, the fluorinated conversion-type cathode pairing with Li metal anode can theoretically provide a specific energy as high as 850 Wh/kg^1,25^. However, it usually suffers from the deactivation and dissolution of conversion products, leading to the F loss and fast capacity degradation^26^. The configuration of SSEs can reinforce the volume compaction effect of conversion products and suppress their extrusion (or dissolution) into electrolyte. For example, ceramic-based solid-state Li||FeF_3_ batteries with improved cycling behavior have been recently reported in the literature^27^. However, the higher capacity release and better cycling durability still require the soft cathode interface contact instead of hard ceramic interface. The stacking of hard C–N polymer microspheres in soft PEO matrix softens the cathodic interface to a certain degree without compromise of anode stability, demonstrating a new avenue for solid-state Li||FeF_3_ batteries^10^. However, the heterogeneity of this composition electrolyte is still evident due to the phase segregation between g-C_3_N_4_ and PEO, leading to the potential coarsing of electrolyte especially after repeated cycling. The F-contained active species is prone to irreversibly migrate into electrolyte due to the driving of F concentration gradient and trapping by heterogeneous grain boundaries, causing the capacity attenuation especially during the early cycles. Based on these considerations, here we propose a dual fluorination of conversion cathode and PEO-based electrolyte to construct the fluoride-integrated solid-state batteries. This fluorinated solid electrolyte is prepared by introducing nanoparticles of mesoporous α-aluminum fluoride with high-specific surface area (denoted as HS-AlF_3_) into PEO matrix for the first time (Fig. 1). This HS-AlF_3_ has the strong Lewis acidity^28^, and can accelerate the dissociation of adjacent lithium-based salt and enhance the overall transport of lithium ions. The HS-AlF_3_ synthesized by a sol-gel method is rich in C, O, N-bonded species at particle surface, and these organic groups can promote its crosslinking with PEO. The mesoporous morphology of HS-AlF_3_ constructed by the self-assembly of primary particles can guarantee its multi-scale interaction with Li salts and PEO segments. More importantly, this well-dispersed filler with high F content can improve the homogeneity of composition electrolyte with homogeneous distribution of F element, which can provide the F-concentrated smooth interface to mitigate the F-loss from the fluoride cathode. These factors guarantee the achievement of effective Li–Fe–F conversion-based solid-state batteries even under the thin electrolyte membrane thickness (45 μm) and lab-scale single-layer pouch cell configuration. This highly fluorinated polymer electrolyte also enable the stabilization of Li metal anode and high-voltage cathode during long-term cycling.

## Results

### Synthesis and physicochemical characterization of HS-AlF_3_

**Fig. 2:**
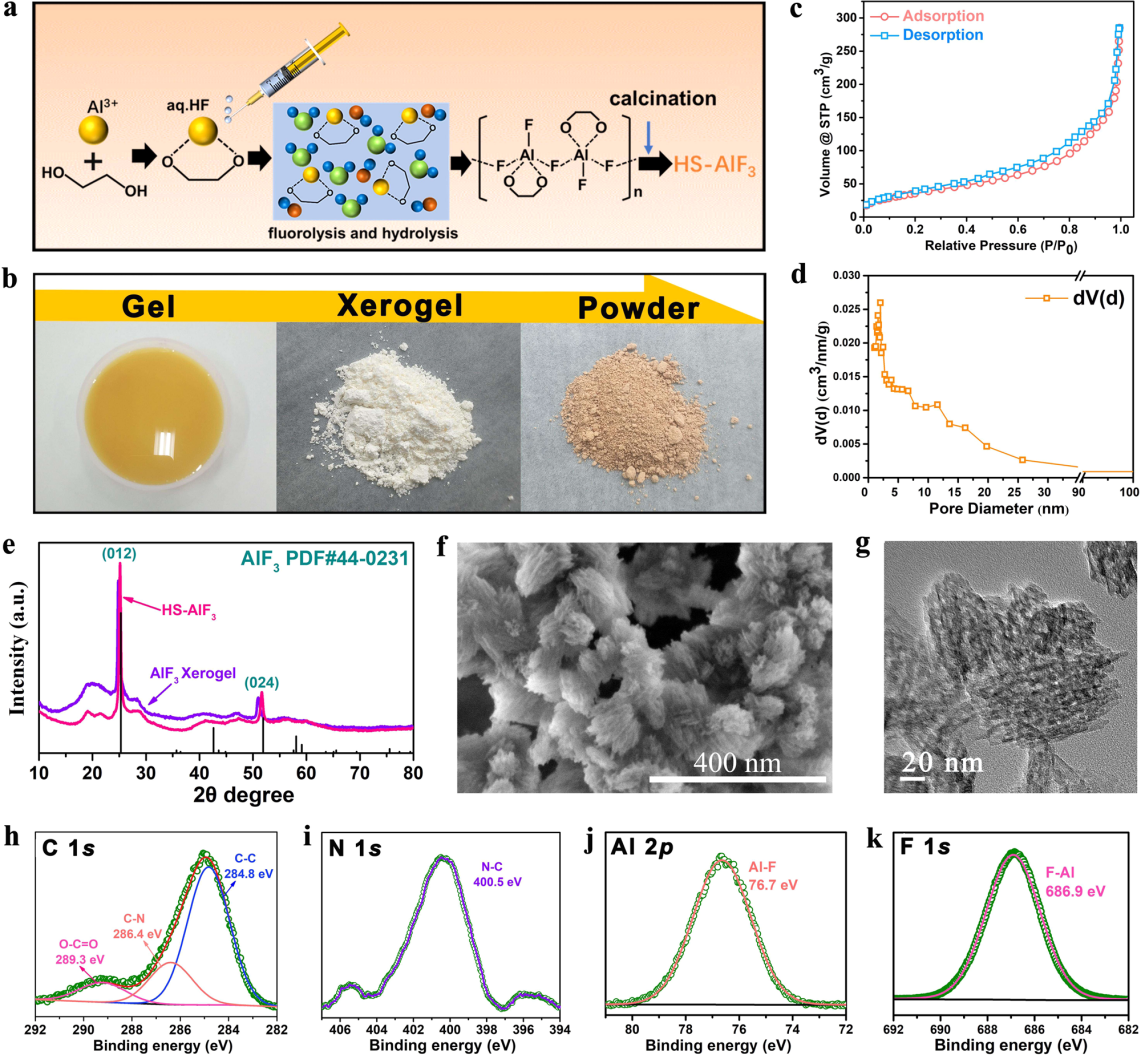
Synthesis and characterization of HS-AlF_3_. **a** Schematic synthesis process of HS-AlF_3_. **b** Photos of wet gel, xerogel and final powder in the fluorolytic sol-gel route for HS-AlF_3_. **c** Nitrogen sorption isotherms of HS-AlF_3_ powder and (**d**) BJH pore size distribution from the adsorption branch. **e** XRD patterns of AlF_3_ xerogel and HS-AlF_3_ powder. **f** SEM and (**g**) TEM images of as-prepared HS-AlF_3_ powder. XPS spectra of HS-AlF_3_ powder with the signals of (**h**) C 1*s*, (**i**) N 1*s*, (**j**) Al 2*p* and (**k**) F 1*s*.

Figure 2a shows the schematic synthesis process of HS-AlF_3_. The fluorolytic sol-gel route plays an important role in the preparation of nanostructured metal fluorides with high-specific surface area^29^. Commercially low-cost Al(NO_3_)_3_·9H_2_O was used as aluminum source, and ethylene glycol (EG) with the strong chelating ability with Al ions was used as solvent. Their mixture was stirred at 50 °C to form a saturated solution of metal alkoxide. Then a dilute aqueous hydrofluoric acid (40 wt%) with stoichiometric ratio was added as F source. The fluorination and hydrolysis processes progressed during the following stirring for 6 h. The wet gel with a light-yellow color was obtained after the aging process for 24 h at 90 °C and then it was dried at 120 °C for another 24 h to form a white xerogel powder (Fig. 2b). Afterwards, a light HS-AlF_3_ powder with a brown color was achieved after a high-temperature calcination (400 °C) of the above xerogel intermediate. Based on this technology process, the HS-AlF_3_ filler product as much as 8 g can be synthesized for one time (Supplementary Fig. 1). Figure 2c, d reveals a high Brunauer–Emmett–Teller (BET) specific surface area of 188 m^2^/g for the obtained AlF_3_ by the N_2_ sorption measurement. The pore size is estimated from the isotherm adsorption branch by using the Barrett-Joyner-Halenda (BJH) model. The pore volume is 0.44 cm^3^/g and the pore radius range is around 2–13 nm. Compared with the other methods using different precursors (e.g. fluorinated metal hydrazine and F_2_ gas), this sol-gel method can achieve the higher specific surface area and pore volume for porous AlF_3_^30^. Both the xerogel and HS-AlF_3_ samples present two significant X-ray diffraction (XRD) peaks at 25° and 52° (Fig. 2e), corresponding to the α-AlF_3_ phase (JCPDS #44-0231). The AlF_3_ xerogel is sintered in air in order to remove the organic species on its surface and obtain the characteristics of high-specific surface area. However, this process may be accompanied by superficial oxidation of sample, so there may be a small amount of Al-O-F or Al-O compounds (also with Lewis acidic nature) in the surface layer, which corresponds to the small peaks around 20° in the XRD pattern. These weak impurity phases would not play an important role in the interaction with the polymer matrix, and do not affect the performance of the main phase AlF_3_. Note that this fluorolytic sol-gel route causes a preferential crystallization orientation along the (012)/(024) crystal planes. Both the XRD patterns also show a peak shift towards low angles, which is likely caused by the nano-sizing and defect doping of AlF_3_ particles. The xerogel without annealing treatment shows a greater deviation of diffraction peaks and the crystallinity for HS-AlF_3_ is enhanced with a smaller deviation of peak positions after sintering. Since the organic layer in AlF_3_ xerogel is gradually lost during sintering in air, the nanocrystalline grains of AlF_3_ grow slightly and at the same time more effective Lewis acid sites in this solid acid are exposed. It is favorable for the better interaction with PEO-based electrolyte components^29^. The scanning electron microscopy (SEM) image of as-prepared HS-AlF_3_ discloses the ordered agglomeration of numerous nanorods to promote the formation of mesoporous morphology (Fig. 2f and Supplementary Fig. 2). This nanocluster texture is further revealed by transmission electron microscopy (TEM) imaging (Fig. 2g and Supplementary Fig. 3), where the typical length of AlF_3_ particles is around 50–80 nm and the diameter is no more than 10 nm. The HS-AlF_3_ is featured by its high Lewis acidity, which makes it a promising solid catalyst. The acidic surface centers are expected to stem from the coordination-unsaturated aluminum atoms^28^. The smaller particles have a higher fraction of surface atoms and five-fold and even four-fold coordinated aluminum species than large particles^31^. So, these HS-AlF_3_ nanocrystals enable the exposure of more Lewis acid sites, leading to a more sufficient interaction with C–O–C segments, which are considered as Lewis base centers in PEO chains^5^. Besides, the pillaring of these thin AlF_3_ nanoparticles with a high aspect ratio among PEO chains is beneficial for the amorphization of PEO microscopic areas. The X-ray photoelectron spectra (XPS) of C 1*s* and N 1*s* confirm the grafting of organic moieties on the HS-AlF_3_ surface (Fig. 2h, i). The C–C (284.8 eV), C–N (286.4 eV), and O–C=O (289.3 eV) peaks are observed in C 1*s*, and the C–N bonding (400.5 eV) is observed in N 1*s*^32^. The residual of carbon and nitrogen contents should come from the Al-F-linked polymer intermediate with potential interaction with NO_3_^−^ anion after mild pyrolysis process (Fig. 2a). The dominant Al-F peaks at 76.7 eV in Al 2*p* and at 686.9 eV in F 1*s* denote the AlF_3_ phase (Fig. 2j, k)^33^. The wrapping of organic components can promote the molding of high-surface mesoporous texture in AlF_3_. It prevents the merging of AlF_3_ nanoparticles into large chunks with the undesired loss of Lewis acid sites.

### Physicochemical and electrochemical characterizations of fluorinated polymer electrolyte

**Fig. 3:**
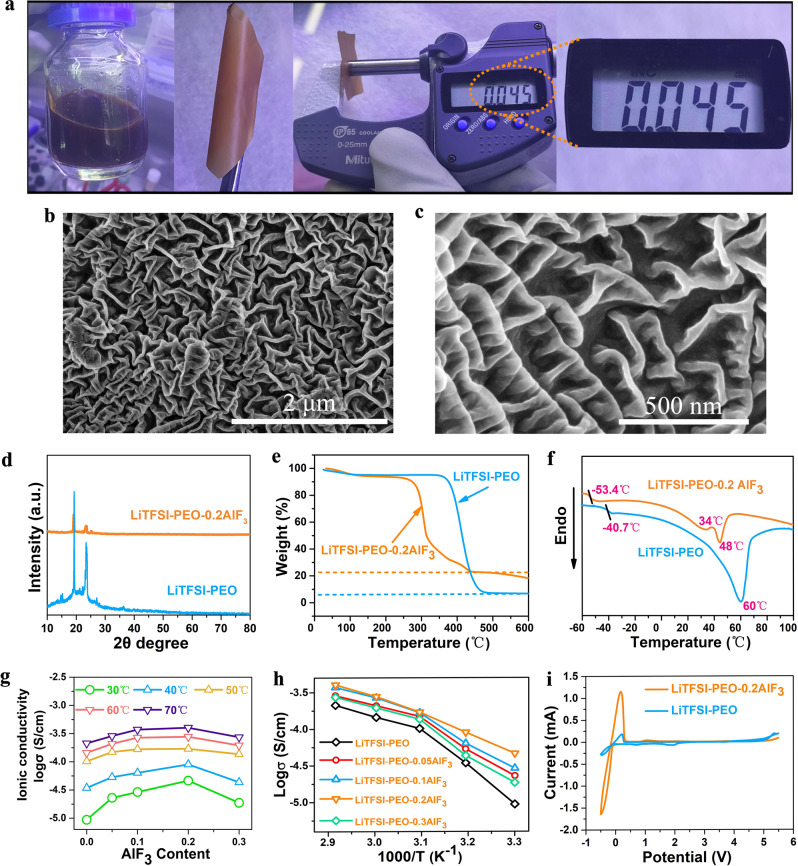
Morphology, thermal and conductivity properties of fluorination-reinforced polymer electrolyte. **a** Photos of reddish-brown solution containing HS-AlF_3_ powder, LiTFSI and PEO, as well as AlF_3_ reinforced composite polymer membrane with a thickness of 45 μm. **b**, **c** SEM images of composite polymer electrolyte of LiTFSI-PEO-0.2AlF_3_ at various magnifications. **d** XRD patterns of LiTFSI-PEO-0.2AlF_3_ and LiTFSI-PEO. **e** TGA and (**f**) DSC curves of LiTFSI-PEO-0.2AlF_3_ and LiTFSI-PEO membranes. **g** Ionic conductivity measurements of solid polymer electrolytes by two-electrode Swagelok-type SS|LiTSFI-PEO-AlF_3_|SS cell configuration with different amounts of HS-AlF_3_ filler in different testing temperatures ranging from 30 to 70 °C. **h** Arrhenius plots of corresponding AlF_3_-reinforced composite membranes. **i** CV curves of Li|LiTFSI-PEO-0.2AlF_3_|SS and Li|LiTFSI-PEO|SS cells at a scan rate of 0.5 mV/s at 60 °C.

In a preparation process of the polymer membrane, the brownish HS-AlF_3_, lithium bis(trifluoromethanesulfonyl)imide (LiTFSI) and PEO powders were added into the acetonitrile solution in a certain proportion (see Methods section for details), and then a uniform reddish-brown solution was formed after stirring for several hours. Due to the ordered mesoporous structure of HS-AlF_3_, the solution results to be sticky without bubbling phenomenon during the solution process. The solution was casted on a polytetrafluoroethylene petri dish, and then acetonitrile was completely evaporated in a vacuum environment at 60 °C. The resultant membrane was peeled off, and a brownish flexible polymer electrolyte was obtained. This composite electrolyte is bendable without any naked-eye visible fracture and its thickness can be controlled in as thin as 45 μm. This thickness is also verified from the cross-sectional SEM morphology measurement of electrolyte membrane (Supplementary Fig. 4). Figure 3a shows the corresponding photos of HS-AlF_3_ contained solution as well as the electrolyte membrane. Benefitting from the aerogel-like feature of HS-AlF_3_ and potential multi-scale interaction, this highly fluorinated polymer electrolyte is expected to be robust during the slipping and thinning processes^9^. As a result, our electrolyte membrane can be made thinner, e.g. down to 25 μm, still without fracture even under the high bending state (Supplementary Fig. 5). The surface of HS-AlF_3_ reinforced electrolyte shows a corrugated morphology with the appearance of uniform sheet-like protrusions with a thickness of less than 50 nm (Fig. 3b, c). This surface texture is likely attributed to the crosslinking and contact between HS-AlF_3_ and LiTFSI-PEO. This granular-free morphology indicates the potential dissolution effect of HS-AlF_3_ in the PEO matrix, which promotes the homogeneity of composite electrolyte and the penetration of crosslinking interfaces. This phenomenon is different from the discrete grain distribution of traditional fillers (e.g. Al_2_O_3_ and ZrO_2_), which localizes the contact interfaces with LiTFSI-PEO and therefore the conduction region^8^. The energy dispersive spectra (EDS) mapping further confirms the dispersion effect of HS-AlF_3_ in the polymer matrix (Supplementary Fig. 6). The distributions of F and Al elements are uniform, and there is no grain aggregation area. The C and O elements mainly come from PEO, and N mainly comes from the salt anion TFSI^−^, and their distributions are also uniform. Figure 3d shows the comparison of XRD patterns of 20 wt% HS-AlF_3_ filled LiTFSI-PEO electrolyte (denoted as LiTFSI-PEO-0.2AlF_3_) and pure LiTFSI-PEO electrolyte. The pillaring and dissolution of AlF_3_ enable a significant reduction of PEO crystallinity. The (012) diffraction peak of AlF_3_ in LiTFSI-PEO-0.2AlF_3_ is still observable, indicating the incomplete dissolution of fluoride fillers. These buried-in granules are still useful in terms of internal mechanics and interaction reinforcements. Figure 3e shows the thermogravimetric analysis (TGA) of LiTFSI-PEO-0.2AlF_3_ and LiTFSI-PEO. Note that the AlF_3_ hybridization reduces the decomposition temperature of the electrolyte system, but it is still as high as ~280 °C. Due to the high thermal stability of AlF_3_, the residue of composite polymer electrolyte is still up to 20% even when the temperature is close to 600 °C, and this residue fraction is consistent with the filler content. Figure 3f compares the curves of differential scanning calorimetry (DSC) analysis of LiTFSI-PEO-0.2AlF_3_ and LiTFSI-PEO in the temperature range from −60 °C to 100 °C. The sharp endothermic peak observed at 60 °C corresponds to the melting temperature (*T*_m_) of LiTFSI-PEO. The *T*_m_ value decreases to 48 °C for LiTFSI-PEO-0.2AlF_3_ in view of the potential fluorination of PEO segments by dissolved F-ions^34^. An extra enthalpy peak is observed at lower 34 °C, and it is likely associated with the more sufficient fluorination effect on some –C–O–C– chains. The glass transition temperatures (*T*_g_) of LiTFSI-PEO-0.2AlF_3_ and LiTFSI-PEO are −53.4 °C and −40.7 °C, respectively. The lower *T*_g_ for LiTFSI-PEO-0.2AlF_3_ indicates that the pinning of AlF_3_ particles with a strong plasticizing effect can further reduce the crystallinity of LiTFSI-PEO, which is beneficial to the conductivity enhancement of composite electrolyte.

In order to estimate the optimization effect of Li-ion conductivity by AlF_3_ filling, we conducted a series of electrochemical impedance spectroscopy (EIS) measurements based on the configuration of SS|LiTFSI-PEO-*x*AlF_3_|SS (SS denoting stainless steel) in a frequency range of 10^7^−0.01 Hz at 30–70 °C. Figure 3g shows the conductivity varieties of LiTFSI-PEO-*x*AlF_3_ as a function of AlF_3_ content at different temperatures. The formula of LiTFSI-PEO-0.2AlF_3_ has the highest conductivity, which is 4.63 × 10^−5 ^S/cm at 30 °C and is improved to 2.78 × 10^−4 ^S/cm at 60 °C (Supplementary Fig. 7 and Supplementary Table 1). This optimized fraction of HS-AlF_3_ (20 wt%) is higher than other reports where the optimal contents of inert fillers are often around 10 wt%^11,35,36^. The higher fraction exposure of cross-linkable sites in HS-AlF_3_ is responsible for the delaying saturations of fluorinated interfaces and amorphized PEO domains. Therefore the higher content of fluoride filler with even cluster- or molecule-scale interaction still takes effect on the further improvement of ionic conductivity performance. Figure 3h shows the Arrhenius plots of AlF_3_ integrated electrolytes based on different filler contents. Note that there are turning points (around 50 °C) in the plots of LiTFSI-PEO-*x*AlF_3_ (*x* = 0, 0.05, 0.1, and 0.3) in the cases of insufficient and excess HS-AlF_3_ filling. For these non-optimized electrolytes, the activation energy is high (~0.83 eV) below the turning point, indicating a low Li-ion conductivity in the temperature range below 50 °C. When the testing temperature steps across the melting point of polymer (48 °C), the activation energy changes to a small value (0.33 eV) and the diffusion behavior of Li-ions is activated. In contrast, the Arrhenius plots for optimized LiTFSI-PEO-0.2AlF_3_ can nearly comply with a linear relationship in the whole temperature range, showing an activation energy of 0.48 eV. Therein the high ionic conductivity behavior can be extended to the lower temperature zone. The increase of ionic conductivity should be attributed to the penetrations of amorphized PEO domains and fluorinated interfaces where the high-speed movement of Li-ions is not disrupted.

In order to confirm the reinforcement effect of electrochemical stability in the AlF_3_-composited electrolyte, we conducted the cyclic voltammetry (CV) measurements based on the asymmetric configuration of Li|LiTFSI-PEO-0.2AlF_3_|SS and Li|LiTFSI-PEO|SS cells (Fig. 3i). Note that both the cells experience a slight oxidation near 5 V. The LiTFSI-PEO-based cell shows an earlier oxidation disorder at ~ 4.6 V and therein the larger current response indicates its worse electrochemical stability at high voltage. The AlF_3_ infiltrated electrolyte membrane is stable up to 4.9 V during the positive scanning with a smaller current response due to its better high voltage tolerability. During the Li plating and stripping at the low voltage side, both the cells experience similar reduction potentials from 0 to −0.5 V. However the Li|LiTFSI-PEO-0.2AlF_3_|SS cell has the smaller oxidation and reduction overpotentials than those for Li|LiTFSI-PEO|SS. Moreover both the plating and stripping peaks for LiTFSI-PEO-0.2AlF_3_ are more symmetric and much sharper compared with those for LiTFSI-PEO. The much more prominent current response peaks should be caused by the higher conductivity of LiTFSI-PEO-0.2AlF_3_ separator and its better anode stability towards Li metal^37^. We also conducted the electrochemical floating experiment to verify the widened electrochemical window based on the cell configuration of Li|LiTFSI-PEO-0.2AlF_3_|LiNi_0.8_Co_0.1_Mn_0.1_O_2_ (NCM811) (Supplementary Fig. 8). The low current response from 4.3 to 5.3 V indicates the electrochemical stability of AlF_3_ reinforced polymer at high polarization voltages exceeding 5 V, which is favorable for the matching with NCM811 high-voltage cathode as shown later.

### Analysis of Li-ion conduction mechanism

**Fig. 4:**
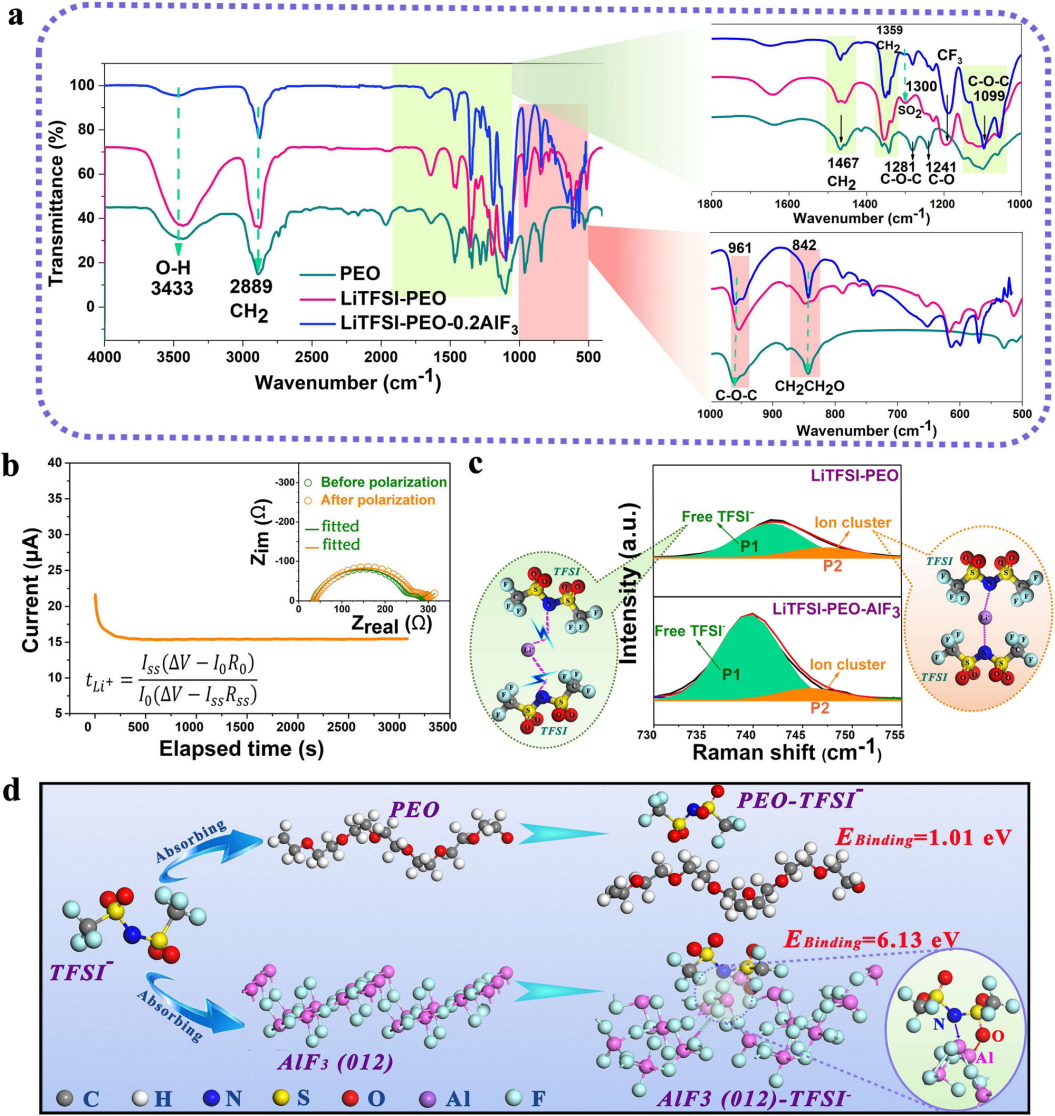
Surface interaction detection between HS-AlF_3_ and PEO or LiTFSI. **a** FTIR spectra of PEO, LiTFSI-PEO and LiTFSI-PEO-0.2AlF_3_. The amplified spectra in the wavenumber ranges of 1000–1800 cm^−1^ and 500–1000 cm^−1^ are shown as insets on the right. **b** Chronoamperometry curve of Li|LiTFSI-PEO-0.2AlF_3_|Li symmetric cell at a voltage bias of 10 mV for a duration time of 3100 s, inset: AC impedance spectra of Li|LiTFSI-PEO-0.2AlF_3_|Li cell before and after polarization at 60 °C. **c** Raman spectra of LiTFSI-PEO and LiTFSI-PEO-0.1AlF_3_ in the wavenumber range of 730–755 cm^−1^ for the comparison of dissociation effect of AlF_3_ on LiTFSI. **d** Schematic illustration showing the structures of PEO chain and AlF_3_ (012) plane, as well as the adsorption binding energies between AlF_3_ and TFSI^−^ and between PEO and TFSI^−^.

Figure 4a shows the comparison of Fourier transform infrared (FTIR) spectra of PEO, LiTFSI-PEO, and LiTFSI-PEO-0.2AlF_3_. After the inclusion of HS-AlF_3_, the vibration peak of hydrogen bonds between PEO chains at 3433 cm^−1^ is weakened^38^. This can be ascribed to the weakening of interchain force and interruption of PEO chains by the potential fluorination of dissolved AlF_3_ and loss of polymer crystallinity^39^. The filling of nano-AlF_3_ also influences the vibrations of CH_2_ in view of the potential formation of C–F. Compared with LiTFSI-PEO, the peaks at 2889 cm^−1^ and 1467 cm^−1^, respectively corresponding to the symmetric stretching and bending vibrations of CH_2_, are weakened^40^. However, the vibration of CH_2_ at 1359 cm^−1^ is enhanced due to the strong vibrations of C–O–C groups and their linkage effect. The unusual enhancement of vibration intensity at 1099 cm^−1^ (belonging to the symmetric stretching of C–O–C) should be triggered by the strong interference by Lewis acid centers of AlF_3_^31^. The enhanced vibration peaks at 961 cm^−1^ and 842 cm^−1^, which correspond to the deformation vibrations of C–O–C and CH_2_CH_2_O respectively, also support this fluorination interference mechanism^41^. This FTIR result indicates the strong interaction of HS-AlF_3_ with PEO chains, which promotes the high-speed oscillation of C–O–C groups and favors for the establishment of high-conductivity interfaces between polymer chains and fluoride lattices.

In order to detect the influence of HS-AlF_3_ on the *t*_Li+_ value of polymer electrolyte, we conducted the combined chronoamperometry and alternating-current (AC) impedance measurements based on the Li|LiTFSI-PEO-*x*AlF_3_|Li cell configuration. The *t*_Li+_ value is obtained according to the equation (Fig. 4b and Supplementary Table 2)^42^:

1 $${t}_{{{{{{{\rm{Li}}}}}}}^{+}}=\frac{{I}_{{{{{{\rm{ss}}}}}}}(\triangle V-{I}_{0}{R}_{0})}{{I}_{0}(\triangle V-{I}_{{{{{{\rm{ss}}}}}}}{R}_{{{{{{\rm{ss}}}}}}})}$$

where Δ*V* is the bias voltage (10 mV), *I*_0_ and *I*_ss_ are the polarization currents of initial and steady states respectively, and *R*_0_ and *R*_ss_ are the cell resistances before and after DC polarization respectively. The *t*_Li+_ value is calculated to be as high as 0.67 for the optimized LiTFSI-PEO-0.2AlF_3_, which is nearly threefold that of reported LiTFSI-PEO electrolyte at 60 °C (0.13–0.25)^43^. The nano-AlF_3_ with high Lewis acidity is expected to bind the TSFI anions and retard their migration, and therefore contribute to the high *t*_Li+_. Insufficient or excessive filling of AlF_3_ would limit the effect of acidity or block the Li-ion transport channels, thus still leading to the low *t*_Li+_ values. (Supplementary Fig. 9 and Supplementary Tables 3–5). In order to indicate the origin of high lithium transference number, we conducted the Raman spectroscopy measurements of LiTFSI-PEO and LiTFSI-PEO-0.1AlF_3_ (Fig. 4c). Since the Raman signals cannot be detected for LiTFSI-PEO-0.2AlF_3_ sample due to the strong fluorescence effect, we intentionally lowered the filler content to 10 wt% for the successful measurement. According to the previous report by Chen et al.^44^, there are two main states referring to TFSI^−^ anion in PEO-based electrolyte, i.e. the disassociated (uncoordinated) TFSI^−^ and ion cluster (two TFSI^−^ coordinated with Li^+^). The Raman peaks at 740–744 cm^−1^ corresponding to disassociated TFSI^−^ state are denoted as P1 band, and those at 747–750 cm^−1^ corresponding to cluster state as P2 band. Assuming that the scattering cross-sections for disassociated TFSI^−^ and ion cluster are similar, the former percentage is estimated according to the equation: [TFSI^−^] = *A(*P1)/[*A*(P1) + *A*(P2)] × 100%, where A(P1) and A(P2) are the integrated areas of P1 and P2 bands respectively. The percentage of disassociated TFSI^−^ in LiTFSI-PEO is estimated to be 77%, indicating the existence of redundant ion clusters. In LiTFS-PEO-0.1AlF_3_, the area fraction of P1 band is enhanced and the percentage of disassociated TFSI^−^ reaches 89%, proving the better splitting of Li-[TFSI]_n_ ion clusters triggered by the fluorination of Lewis acid. In order to verify the adsorption effect of AlF_3_ surface towards TFSI^−^ anion, we used density functional theory (DFT) calculations to compare the binding energies between TFSI^−^ and PEO and between TFSI^−^ and dominant (012) plane of AlF_3_ (Fig. 4d). For the TFSI^−^-PEO system, the oxygen atoms in TFSI^−^ are close to the hydrogen atoms in PEO chain, leading to adsorption energy of 1.01 eV between PEO and TFSI^−^. For the TFSI^−^-AlF_3_ system, the oxygen and nitrogen atoms in TFSI^−^ are strongly attracted by the centric Al atoms on the AlF_3_ surface. The adsorption energy between AlF_3_ and TFSI^−^ is as high as 6.13 eV, indicating preferential adsorption of TFSI^−^ by AlF_3_ surface and therefore the liberation of TFSI^−^ from Li-[TFSI]_n_.

### AlF_3_ fluorination effect on the stabilization of Li anode

**Fig. 5:**
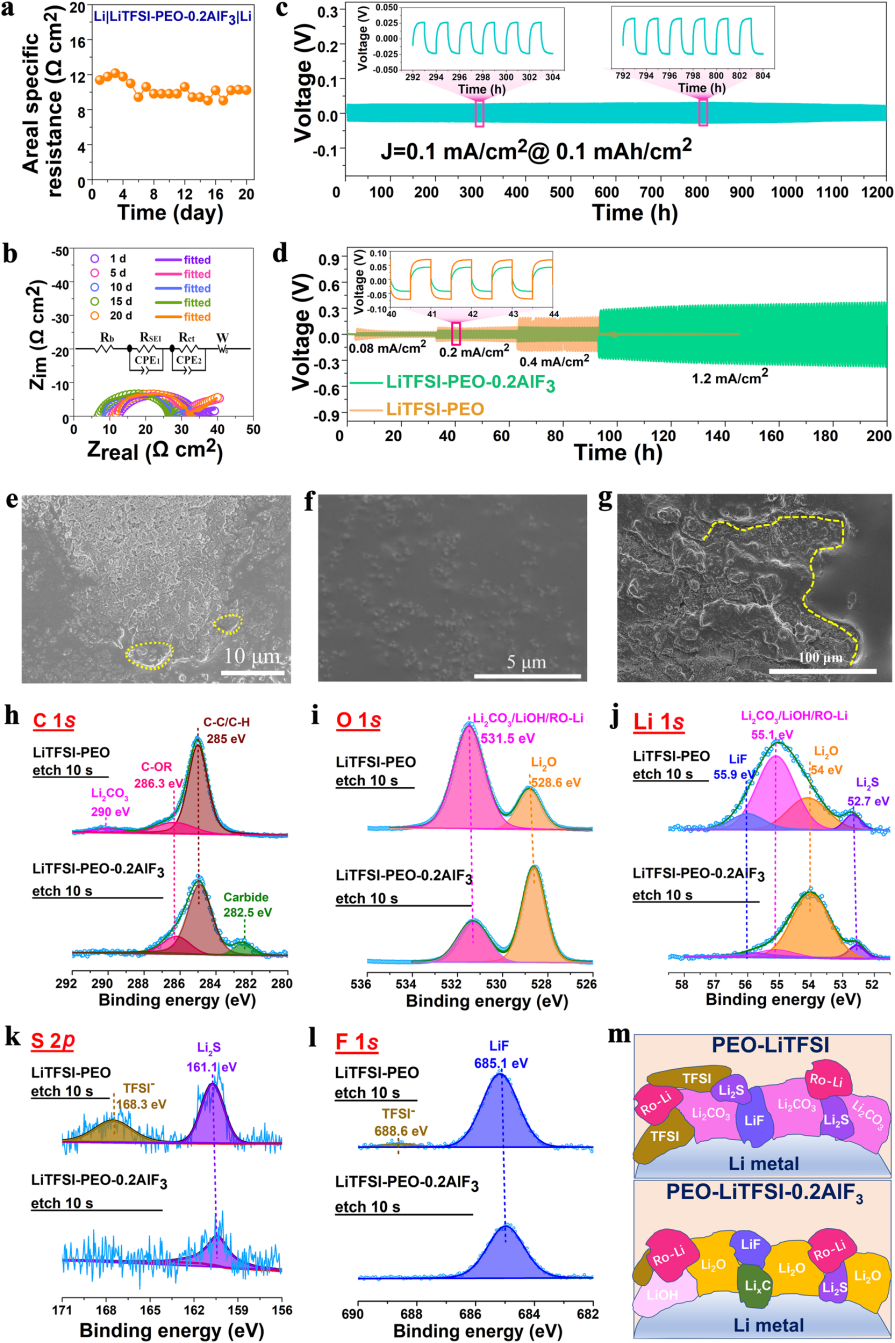
Anode–electrolyte interface stability, morphology and component evolution during Li plating/stripping. **a** Areal specific resistance evolution as a function of aging time based on Li|LiTFSI-PEO-0.2AlF_3_|Li symmetric cell. **b** Corresponding Nyquist plots of the EIS measurements at different evolution stages, where d refers to day(s). **c** Galvanostatic Li plating/stripping curves of Li|LiTFSI-PEO-0.2AlF_3_|Li symmetric cell as a function of cycling time at 0.1 mA/cm^2^, inset: magnified curves at different cycling stages. **d** Comparison of Li plating/stripping cycling processes for Li|LiTFSI-PEO-0.2AlF_3_|Li and Li|LiTFSI-PEO|Li symmetric cells at different current densities, inset: magnified curves at 0.2 mA/cm^2^. **e**–**g** Ex situ SEM images of lithium metal anode after cycling of Li|LiTFSI-PEO-0.2AlF_3_|Li symmetric cell for 300 h at 0.1 mA /cm^2^ in different regions. Ex situ XPS measurements of lithium metal anode surface after cycling for 120 h for both the LiTFSI-PEO-0.2AlF_3_ and LiTFSI-PEO systems, with the signals of (**h**) C 1*s*, (**i**) O 1*s*, (**j**) Li 1*s*, (**k**) S 2*p* and (**l**) F1*s*. **m** Schematic illustration of components at the surface of cycled lithium metal anode based on LiTFSI-PEO and LiTFSI-PEO-0.2AlF_3_.

Figure 5a, b shows the areal specific resistance (ASR) values of Li|LiTFSI-PEO-0.2AlF_3_|Li symmetric cell and corresponding impedance spectra at different calendar aging stages. The observed semicircle size (Fig. 5b and Supplementary Table 6) denotes the interfacial resistance (including the contributions from charge transfer and solid electrolyte interphase SEI)^45^. Note that, in Fig. 5a, the interfacial ASR value is quite stable and stands at a value of about 10 Ω cm^2^ (based on the unilateral interface) for at least 20 days. The mitigation of passivation side reactions is responsible for the stability of anode–electrolyte interface with small ASR value, which is favorable for the long-term cycling of Li plating and stripping processes of Li|LiTFSI-PEO-0.2AlF_3_|Li symmetric cells even based on an electrolyte membrane as thin as 45 μm (Fig. 5c). The stable plating and stripping processes can last at least 1200 h at 0.1 mA/cm^2^ and 0.1 mAh/cm^2^ with a unilateral overpotential as small as 25 mV, which is about half of the previous reports based on other fillers at the similar current density conditions^10,11^. Note that the overpotential slightly decreases with the increase of cycling time, and it can be ascribed to the further decreased interfacial resistance as shown in Supplementary Fig. 10 and Supplementary Table 7. After the cycling for 200 h, the ASR value is decreased to 7 Ω cm^2^. When doubling the current density and areal capacity to 0.2 mA/cm^2^ and 0.2 mAh/cm^2^ respectively, the Li|LiTFSI-PEO-0.2AlF_3_|Li cell still enables a stable electrochemical cycling for at least 560 h and a low overpotential of ~50 mV (Supplementary Fig. 11). Figure 5d shows the comparison of rate performances based on LiTFSI-PEO-0.2AlF_3_ and LiTFSI-PEO electrolytes. At the larger current density of 0.4 mA/cm^2^, the voltage polarization of Li|LiTFSI-PEO-0.2AlF_3_|Li cell is half of that of Li|LiTFSI-PEO|Li. At 1.2 mA/cm^2^, the fluorination-modified electrolyte enables a stable cell operation for more than 100 h with an overpotential smaller than 300 mV, whereas the LiTFSI-PEO-based cell undergoes a sharp short-circuit. Via ex situ SEM measurement pores and cracks can be observed on the LiTFSI-PEO membrane surface (Supplementary Fig. 12), and these undesired regions may further deteriorate during electrochemical cycling. This morphology is caused by the poor mechanical properties of LiTFSI-PEO, which is not effective to suppress Li dendrite formation, therefore leading to the earlier short circuit and quicker capacity degradation. Supplementary Fig. 13 and Supplementary Table 8 show the ASR values of Li|LiTFSI-PEO|Li cell and corresponding EIS evolution after different aging times. The resistance value increases with the aging time, e.g. from 10.5 Ω cm^2^ after one day to 22.5 Ω cm^2^ after 14 days. The larger and unstable interface resistance further indicates the poor contact stability of LiTFSI-PEO with Li metal with potential interface passivation. This fluorination modulation method enables reduced interface resistance and overpotential for polymer electrolytes, which have the advantages over previous reports based on other filler modifications (Supplementary Fig. 14)^10,11,35,36,45–50^.

Figure 5e, f shows the ex situ SEM images of Li metal surface after cycling for 300 h. The anode surface is overall dense and smooth even after long-term cycling, although some granular Li regions appear. These Li granules are compactly deposited without the generation of porous structures or Li dendrites. Note that some Li surface regions are still wrapped by residual polymer components. This tight and conformal contact between Li metal and solid electrolyte is responsible for the suppression of Li mass extrusion. The formation of fluorinated “interface fusion layer” (marked areas in yellow dotted circles) during electrochemical cycling is favorable for the homogenization of Li-ion flow and mitigation of mechanical damage to avoid the tearing phenomenon of the interface layer. The “interfacial fusion layer” should be caused by the Li extrusion/extraction process, which promotes the shallow penetration and infusion of polymer moieties in pit sites during electrochemical cycling (Fig. 5g and Supplementary Fig. 15). This effect heals the voids between Li and polymer and promotes the transport performance of Li-ion and Li mass at the interface, consistent with the interfacial resistance decrease after cycling (Supplementary Fig. 10). This “interfacial fusion layer” is not caused by the side reaction between LiTFSI-PEO-0.2AlF_3_ film and Li metal, since the interfacial passivation does not occur from the highly stable interface resistance with the aging time (Fig. 5a) and the interface activation during electrochemical cycling (Supplementary Fig. 10). In order to detect the components of cycled Li surface, we conducted the ex situ XPS measurements of the Li metal electrodes as shown in Fig. 5h–l. The C1*s* spectrum from the LiTFSI-PEO system consists of the peaks of Li_2_CO_3_ at 290 eV, C-OR at 286.3 eV and C-C/C–H at 285 eV^51^. However, in the AlF_3_ modified system, the Li_2_CO_3_ peak almost disappears and the C-OR peak is correspondingly reinforced. Meantime another new peak corresponding to carbide appears at 282.5 eV^52^. The attenuation of interfacial Li_2_CO_3_ signal after AlF_3_ modulation is also correspondingly observed at 531.5 and 55.1 eV in O 1*s* and Li 1*s* spectra, respectively^53^. Therein the Li_2_O signal (at 528.6 eV for O 1*s* and at 54 eV for Li 1*s*) increases after the introduction of AlF_3_^53^. One can also note the weakening of TFSI^−^ species peak (at 168.3 eV in S 2*p*) and its lithiated product peaks (Li_2_S and LiF) for the LiTFSI-PEO-0.2AlF_3_ system from Li 1*s* and S 2*p* spectra^54^. The corresponding attenuation of LiF and TFSI^−^ peaks is also indicated from the F 1*s* spectra^52^. These XPS evolutions indicate the interfacial passivation in LiTFSI-PEO system by residual Li_2_CO_3_, trapped TFSI and its decomposition products, agreeing with the occurrence of larger overpotentials during Li plating and stripping. In contrast, the pillaring of AlF_3_ enables the better adsorption of TFSI^−^ anions, which are retarded to diffuse into Li anode. As a consequence, the terminated O atoms in PEO segments near Li metal are prone to be lithiated with the formation of Li_2_O and preservation of C-OR motifs. The alleviated trapping and degradation of TFSI^−^ by Li anode do not provide extra carbon source for the conversion from Li_2_O to Li_2_CO_3_ and therefore are responsible for the enrichment of more ionic conductive Li_2_O as dominant SEI component (rather than passivated Li_2_CO_3_) in LiTFSI-PEO-0.2AlF_3_ system (Fig. 5m)^55,56^. The significant increase of Li_2_O in SEI enhances the Li-ion migration across the anode interface, leading to the much-lowered interface resistance and voltage overpotential even after long-time cycling.

### Assembly and testing of various all-solid-state Li metal batteries

**Fig. 6:**
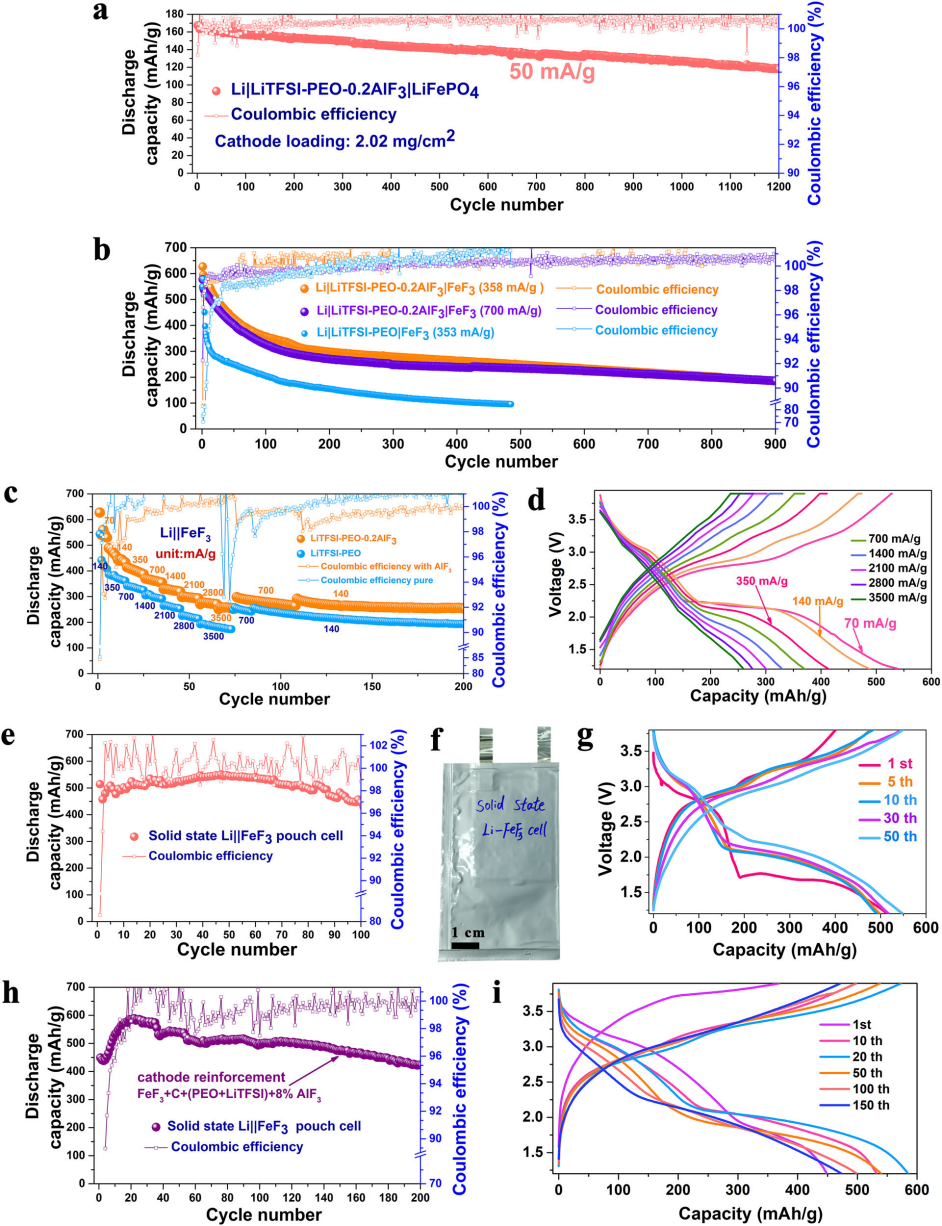
Electrochemical performance of solid-state Li||FeF_3_ cells. **a** Cycling performance of Li|LiTFSI-PEO-0.2AlF_3_|LiFePO_4_ with a cathode loading of 2.02 mg/cm^2^ at 50 mA/g. **b** Cycling performance of Li|LiTFSI-PEO-0.2AlF_3_|FeF_3_ and Li|LiTFSI-PEO|FeF_3_ cells at 358 and 700 mA/g. **c** Rate performance of Li|LiTFSI-PEO-0.2AlF_3_|FeF_3_ and Li|LiTFSI-PEO|FeF_3_ cells. **d** Corresponding charge and discharge profiles of Li|LiTFSI-PEO-0.2AlF_3_|FeF_3_ cell at different rates. **e** Cycling performance of pouch-type all-solid-state Li||FeF_3_ cell based on LiTFSI-PEO-0.2AlF_3_ electrolyte at a specific current of 110 mA/g. **f** Photo of pouch-type Li||FeF_3_ cell. **g** Corresponding charge and discharge curves of pouch-type Li||FeF_3_ cell at different cycling stages. **h** Cycling performance of pouch-type all-solid-state Li|LiTFSI-PEO-0.2AlF_3_|FeF_3_ cell benefiting from cathode reinforcement by AlF_3_ at 153 mA/g. **i** Corresponding charge and discharge curves of cathode-reinforced pouch-type Li||FeF_3_ cell at different cycling stages. The testing temperature is 60 °C and the pressure applied on the pouch cell is 20 KPa, and the specific current and specific capacity refer to the active material’s mass in the positive electrode.

Figure 6a shows the cycling performance of an all-solid-state Li|LiTFSI-PEO-0.2AlF_3_|LiFePO_4_ cell based on a LiFePO_4_ loading of 2 mg/cm^2^ and an electrolyte thickness of 45 μm. The initial discharge capacity is as high as 167.4 mAh/g at 50 mA/g, which is close to the theoretical specific capacity of LiFePO_4_. This thin-lamination polymer membrane enables good cycling stability with the reversible capacities preserved at 141.5 and 126.8 mAh/g after 500 and 1000 cycles respectively. The corresponding charge-discharge curves show a typical voltage plateau of LiFePO_4_ with a small voltage polarization during the whole long-term cycling (Supplementary Fig. 16). This fluorination-reinforced solid-state battery can also display the stable cycling performance at higher rates, e.g. with the capacity preservation at ~100 mAh/g after 3000 cycles under 192 and 317 mA/g (Supplementary Fig. 17). The increase of mass loading of LiFePO_4_ up to 10.2 mg/cm^2^ enables a high area capacity of 1.44 mAh/cm^2^ (138.4 mAh/g) at 18 mA/g during the first cycle (Supplementary Fig. 18a). The reversible capacity is still maintained at 1.22 mAh/cm^2^ (117 mAh/g) after 50 cycles. Such a high mass loading of cathode does not seriously degrade the voltage polarization and plateau feature of LiFePO_4_ (Supplementary Fig. 18b).The fluorinated crosslinking effect broadens the electrochemical window of polymer electrolyte and enhances the oxidation resistance of cathode material^57^. The existence of terminated –OH groups is thought to limit the electrochemical window of PEO electrolyte^58^. The dissolved AlF_3_ may promote the fluorination of terminated groups into C–F or C–O–F. In order to verify the adsorption effect of the AlF_3_ surface towards the terminated PEO chain, we conducted the DFT calculation to study the binding energy between the terminal segment of PEO and dominant (012) plane of AlF_3_ (Supplementary Fig. 19). In the optimized PEO-AlF_3_ structure, the oxygen atoms in the terminated –OH groups are strongly attracted by the F and Al atoms on AlF_3_ surface. The corresponding adsorption energy is as high as 4.826 eV, indicating that the –OH groups at the end of PEO chains are “pinned” by the AlF_3_ filler to form the C–O–Al and C–O–F bonds. These potential interactions are favorable for the distortion and even elimination of free –OH groups and therefore the extension of electrochemical stability window. This makes it possible to optimize the performance of solid-state batteries based on high-voltage NCM811 cathode. As shown in Supplementary Fig. 20, the high reversible cycling of Li|LiTFSI-PEO-0.2AlF_3_|NCM811 is realized in the voltage range of 3–4.2 V, and the reversible capacity remains at 143.7 mAh/g at 96 mA/g after 100 cycles. In contrast, the capacity of corresponding LiTFSI-PEO system decays rapidly during much shorter cycles at 34 and 65 mA/g. In addition, the AlF_3_-reinforced Li||NCM811 cells show the much better rate performance even under high-specific currents of 286 and 572 mA/g.

The HS-AlF_3_ filling and its fluorination crosslinking effect on PEO are favorable for the concentration of F element at the cathode-electrolyte interface, which is expected to suppress the outflowing diffusion of fluorine from cathode to electrolyte when coupled with a fluoride-based positive electrode active material such as FeF_3_ in view of the mitigation of F-concentration gradient across interface. The conversion-type FeF_3_ cathode has high theoretical specific energy and energy density (850 Wh/kg and 1400 Wh/L)^25^, and the abundant iron and fluorine resources further make it appealing as a positive electrode active material for Li-based battery systems. However, during the conversion reaction of lithiated FeF_3_, there are phase transitions with continuous evolution of multiple interfaces^1^. The precipitation and deactivation of fluorinated species likely aggravate the coarsening of product domains and dilution of solid-solid interfaces, which would affect the capacity retention of Li||FeF_3_ electrochemical energy storage system. Moreover, in battery system using nonaqueous liquid electrolyte solutions, the expansion of Li–Fe–F volume and dissolution of iron species would loosen the electronic conduction network of cathode and accelerate the capacity decay^26^. The all solid-state battery architecture with soft and fluorinated interfaces is expected to mitigate these problems of Li||FeF_3_ batteries. The solid-state architecture enables the compaction of conversion products and suppression of active species dissolution^27^. The soft contact between cathode and electrolyte can buffer the mass extrusion and phase boundary migration^59^. The interface fluorination can eliminate the F-concentration gradient and avoid the irreversible trapping of F-contained active species. Therefore the LiTFSI-PEO-0.2AlF_3_ solid electrolyte is expected to be especially effective to ensure the long-term capacity retention of all-solid-state Li||FeF_3_ batteries. Figure 6b shows the cycling performance of Li|LiTFSI-PEO-0.2AlF_3_|FeF_3_ cells at 358 and 700 mA/g. At 358 mA/g, the initial discharge capacity is as high as 630 mAh/g, closing to the theoretical specific capacity of FeF_3_ (712 mAh/g) based on three-electron-transfer reaction^60^. In the next 150 cycles, the discharge capacity undergoes a slow decay in view of the phase transition and phase boundary migration. With the increase of deactivated domains, the spatial distribution and electric contact of conversion products are better stabilized, leading to the release of reversible capacities at 297.6, 248.1 and 201.9 mAh/g after 200, 500, and 800 cycles, respectively. At a higher specific current of 700 mA/g, the capacity evolution is similar without the attenuation trend, and the reversible capacities at 358 and 700 mA/g are comparable after 500 cycles and until 900 cycles. In contrast, the capacity performance of Li|LiTFSI-PEO|FeF_3_ cell is worse, and the discharge capacity declines to 100 mAh/g after 440 cycles. Supplementary Fig. 21 displays the typical two-stage discharge curves where the initial conversion reaction can be identified by the plateau at around 1.8 V. Then both the insertion (with lithium ions reversibly intercalated into the cathode without causing major structural change between FeF_3_ and Li_x_FeF_3_) and conversion reactions are gradually coalesced with the evolution of conversion products and their domain distribution, leading to the appearance of solid-solution-like profiles after long-term cycling. The Li|LiTFSI-PEO-0.2AlF_3_|FeF_3_ cell also exhibits the good rate performance (Fig. 6c, d), and its reversible capacities are 330, 300, 280 and 260 mAh/g at 1400, 2100, 2800 and 3500 mA/g respectively with the average cell discharge voltage of 2.31 V. After returning back to 700 mA/g, the discharge capacity can still recover to 300 mAh/g, indicating the satisfactory high-rate tolerability even for the solid-solid conversion process. As expected, the rate performance is much worse for Li|LiTFSI-PEO|FeF_3_. Note that the phenomenon of early capacity attenuation is absent when operating the solid-state cell directly under the much higher specific current of 1900 mA/g (Supplementary Fig. 22). Instead the capacity activation phenomenon is observed during the early 20 cycles. Its cycling process is quite stable for at least 200 cycles with the reversible discharge capacity as high as 300 mAh/g. This capacity is far away from the theoretical capacity of FeF_3_, meaning a fraction of active material does not participate the lithiation reaction during the one-cycle period due to the delayed electron transport. These unreacted active species can still participate the conversion reaction during the following cycles, responsible for the capacity activation phenomenon. These transient unreacted domains can serve as “buffer stakes” to mitigate the coarsening of reaction products and stabilize the as-formed phase boundaries. They are favorable for the volume confinement of active species. Therefore the better capacity stability is observed under high-rate protocol. The ionic conductivity of this fluorinated polymer also enables the cycling at 30 °C of the Li||FeF_3_ solid-state cells (Supplementary Fig. 23). Its reversible discharge capacity is as high as 350 mAh/g, and the initial capacity attenuation is also avoided when a specific current of 333 mA/g is applied. Its discharge profiles show the identifiable insertion-then-conversion plateaus even under 30 °C condition^61–63^.

We also prepared the electrolyte membrane based on commercial AlF_3_ (denoted as C-AlF_3_) as filler, which is named as LiTFSI-PEO-0.2C-AlF_3_ (i.e. with 20 wt% C-AlF_3_). Note that the corresponding ionic conductivity is lower than that of HS-AlF_3_ reinforced polymer based on the same content of filler (Supplementary Fig. 24a and Supplementary Table 9). The corresponding Li|LiTFSI-PEO-0.2C-AlF_3_|FeF_3_ cell displays an initial discharge capacity as high as 505 mAh/g (Supplementary Fig. 24b, c), but the decay trend is more pronounced than that in the HS-AlF_3_ reinforced case. The former discharge capacity is only 169 mAh/g after 200 cycles. This comparison further confirms the advantage of highly mesoporous AlF_3_ filler, which enables the more uniform distribution of fluorinated interfaces and domains in polymer electrolyte. This effect promotes the interface and cycling stabilities of corresponding solid-state Li||FeF_3_ cells. For the Li|LiTFSI-PEO-0.2AlF_3_|FeF_3_ cells, when increasing the active material loading of cathode to 3 mg/cm^2^ (Supplementary Fig. 25a, b), the first discharge areal capacity is as high as 1.6 mAh/cm^2^, corresponding to a capacity of 549 mAh/g, at a specific current of 67 mA/g. The cell undergoes a stable cycling with the retention of an areal capacity of 1.02 mAh/cm^2^ (347 mAh/g) after 30 cycles. The corresponding charge and discharge profiles of FeF_3_ are still well defined with distinct insertion-conversion plateaus. When further enhancing the mass loading of FeF_3_ active material to 4.2 mg/cm^2^, the first cycle can even deliver a high discharge capacity of 2.2 mAh/cm^2^ (519.4 mAh/g) at a larger specific current of 100 mA/g. This cell also exhibits a stable cycling and maintains an areal capacity of 1.182 mAh/cm^2^ (281.5 mAh/g) after 50 cycles (Supplementary Fig. 25c, d). Note that the increase of mass loading and areal capacity for conversion-type fluoride cathodes is challenging for the development of commercial-type battery products. Our first attempt in this aspect indicates the potentiality of future large-scale application of Li–Fe–F conversion batteries especially based on all-solid-state architecture.

As mentioned before, the synthesis process of HS-AlF_3_ can be effectively employed for scaling-up the production of fluorinated polymer membranes. Therefore we assembled a Li||FeF_3_ pouch-type cell as shown in Fig. 6e–g. This pouch cell can deliver an initial discharge capacity as high as 520 mAh/g at a specific current of 110 mA/g, and the following discharge capacity is well retained at 450–550 mAh/g within 100 cycles. The electrochemical activation phenomenon is also observed as the case of Li||FeF_3_ coin cells tested at high current rates (Supplementary Fig. 22). The corresponding charge and discharge profiles are reversible with the appearance of well-defined two-stage (i.e. insertion stage at ~3 V and conversion stage at ~2 V) discharge plateaus. The satisfactory cycling stability of pouch cells is associated with the incomplete conversion reaction as discussed above. In order to further enhance the ionic conductivity and compensate for the interfacial F loss during cycling, we introduced a small amount (8 wt%) of HS-AlF_3_ to the FeF_3_ cathode, which is wired by PEO-LiTFSI-AlF_3_ (as ionic wires) and carbon additive (as electronic wires) as shown in Supplementary Fig. 26. This fluorinated additive to the cathode network is also expected to promote the fluorinated interface compatibility and integrity between the fluorinated electrolyte and fluorinated cathode. This cathode-reinforced pouch cell displays an increase in terms of reversible capacity and cycling stability (Fig. 6h, i). After 20 cycles, the cell reaches a maximum discharge capacity of 589 mAh/g at 153 mA/g, and the reversible discharge capacity is still preserved at 425 mAh/g after 200 cycles. Considering the average cell discharge voltage and specific discharge capacity of about 2.24 V and 584.5 mAh/g, respectively, at the 20th cycle (see Fig. 6h, i) a specific energy of about 1308 Wh/kg (based on the mass of FeF_3_ active material) can be calculated. The amount of 8 wt% AlF_3_ could be considered as an optimal content from a series of electrochemical performance tests. As a comparison, the cycling performance based on the cathodes with less and excessive HS-AlF_3_ contents (e.g. 3 wt% and 15 wt%) in the pouch-type cell configuration is also shown in Supplementary Fig. 27. For the case of 3 wt% of HS-AlF_3_, the cycling and capacity performances lie between those in the AlF_3_-free and 8wt%-AlF_3_ cases. It further indicates the positive effect of interfacial F supplement (from AlF_3_) on the conversion capacity retention at cathode side. However, the excessive injection of insulating AlF_3_ would interrupt both the ion and electron wires in cathode, and counteract the positive effect of interfacial F supplement. Therefore the corresponding cycling performance becomes worse when increasing the AlF_3_ content to 15 wt% even at low specific currents.

**Fig. 7:**
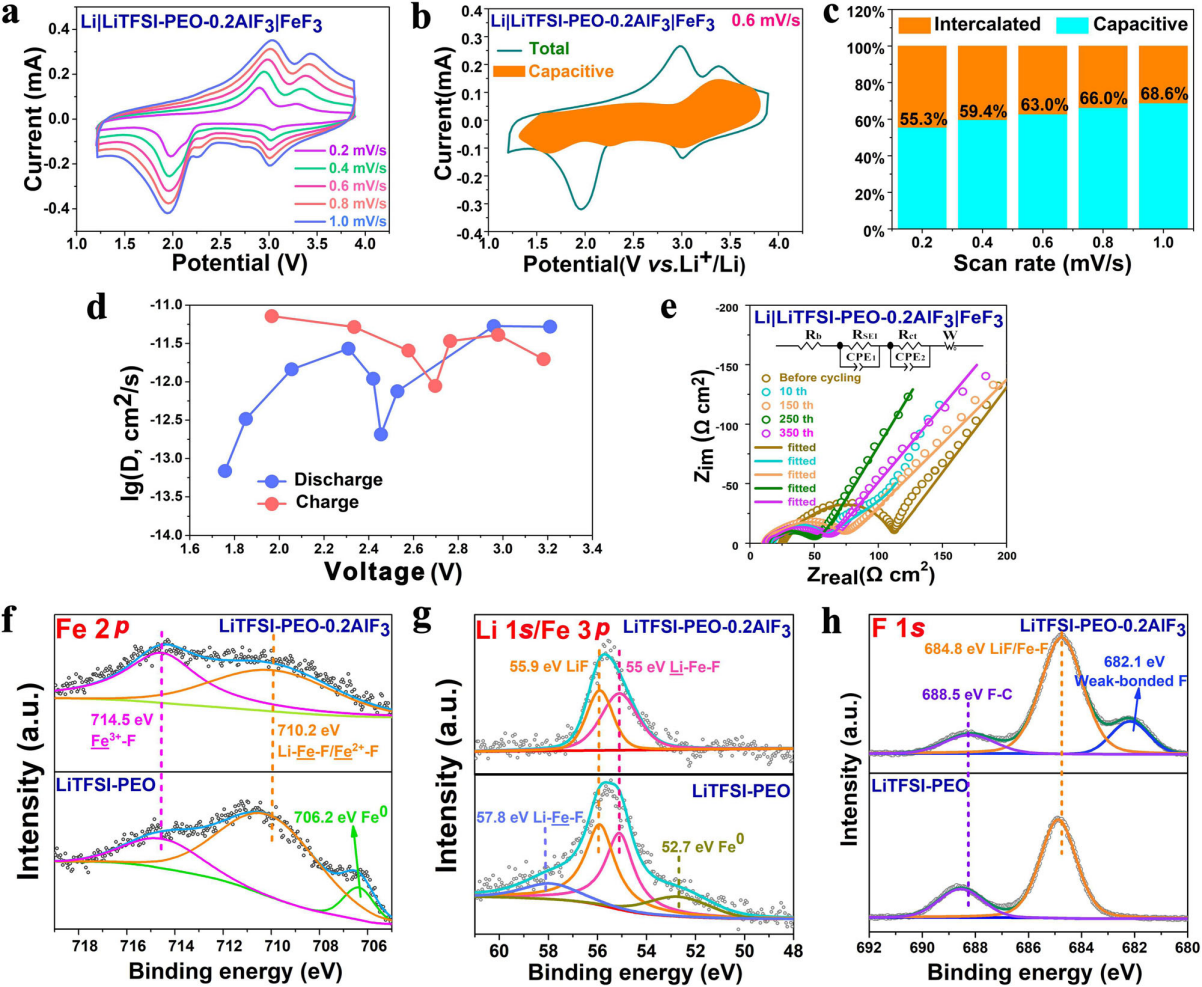
Kinetic performance of fluorinated-polymer-based Li||FeF_3_ cells and surface composition of cathode side. **a** CV curves of Li|LiTFSI-PEO-0.2AlF_3_|FeF_3_ cells at different scan rates from 0.2 to 1 mV/s between 1.2 and 3.9 V. **b** Typical CV curve of Li||FeF_3_ cell at 0.6 mV/s with the capacitive currents (k_1_v) outlined in orange area according to *i*(*V*) = *k*_1_*v* + *k*_2_*v*^1/2^. **c** Histograms of charge storage contributions of capacitance and intercalation processes under different scan rates. **d** Evolution of diffusion coefficient (D) estimated by GITT as a function of cell voltage during discharge and charge processes. **e** Nyquist plots of the EIS measurements of Li|LiTFSI-PEO-0.2AlF_3_|FeF_3_ cells at different cycling stages. In the inset equivalent circuit, *R*_b_ represents the ohmic resistance of cell, *R*_SEI_ represents the lithium diffusion resistance in SEI, and *R*_ct_ presents the charge transfer resistance at the electrolyte-electrode interface. The CPE1 and CPE2 represent the corresponding constant phase elements. W refers to Warburg impedance describing both diffusion and accumulation of Li. Ex situ XPS measurements showing the signals of (**f**) Fe 2*p*_3/2_, (**g**) Li 1 *s/*Fe 3*p* and (**h**) F 1 *s* for the cycled cathodes (FeF_3_ + C + LiTFSI+PEO) at the terminal charging state after 10 cycles based on both the LiTFSI-PEO-0.2AlF_3_ and PEO-LiTFSI electrolytes.

Considering the appealing rate performance of this all-solid-state Li||FeF_3_ cell, we further performed the kinetics analysis by CV and galvanostatic intermittent titration technique (GITT). Figure 7a shows the CV curves of Li|LiTFSI-PEO-0.2AlF_3_|FeF_3_ cell at various scan rates from 0.2 to 1 mV/s. The two-stage redox peaks are recognizable during both the cathodic and anodic processes. The peak positions and intensities agree well with the plateau voltages and lengths in the galvanostatic measurement. We used the equation *i*(*V*) = *k*_1_*v* + *k*_2_*v*^1/2^ to estimate the contribution of pseudocapacitance^64^. Therein i(V) is the current response (i) as a function of voltage (V), k_1_v and k_2_v^1/2^ represent the capacitive and intercalation current intensities, v is the scanning rate. The parameters k_1_ and k_2_ are determined by the linear relationship between i(v)/v^1/2^ and v^1/2^ according to the transformed equation *i*(*V*)/*v*^1/2^ = *k*_1_*v*^1/2^ + *k*_2_. Based on this equation, the capacitive current can be distinguished from the total current, and the pseudocapacitance contribution is disclosed by the enclosed circle (orange area) on the CV curve (Fig. 7b). The high fraction of pseudocapacitance contribution (from 55.3% at 0.2 mV/s to 68.6% at 1.0 mV/s) is responsible for the high-rate performance of Li|LiTFSI-PEO-0.2AlF_3_|FeF_3_ cell (Fig. 7c). The GITT measurement was used to estimate the diffusion coefficient of Li (D) in FeF_3_ cathode. Before GITT, the cell is cycled for 10 cycles at 100 mA/g in order to reach a reversible state. Then the cell runs intermittently for 1 h (3600 s) at 35 mA/g followed by a relaxation process for 6 h in an open-circuit state. The D value can be calculated by the following equation based on the second Fick’s law^65^:

2 $$D=\frac{4}{\pi }{\left(\frac{{m}_{{{{{{\rm{B}}}}}}}{V}_{{{{{{\rm{m}}}}}}}}{{M}_{{{{{{\rm{B}}}}}}}S}\right)}^{2}{\left(\frac{{\triangle E}_{{{{{{\rm{S}}}}}}}}{\tau ({{{{{\rm{d}}}}}}{E}_{{{{{{\rm{\tau }}}}}}}/{{{{{\rm{d}}}}}}\surd \tau )}\right)}^{2}\left(\tau \,\ll \,{L}^{2}/D\right)$$

where *m*_B_, *M*_B_ and *V*_m_ denote the mass, molecular weight (112.8 g/mol) and molar volume (3.56 × 10^−5^ m^3^/mol) of FeF_3_ cathode respectively (calculated from Supplementary Fig. 28), L and S denote the thickness and area of positive electrode respectively, τ is the intermittent time (3600 s), Δ*E*_S_ is the difference of open-circuit voltages after two adjacent relaxation processes. We define Δ*E*τ as the voltage difference during one titration step at 35 mA/g. If the transient potential (Eτ) is linearly related to the square root of τ (Supplementary Fig. 29), the above equation can be simplified as

3 $$D=\frac{4}{\pi \tau }{\left(\frac{{m}_{B}{V}_{m}}{{M}_{B}S}\right)}^{2}{\left(\frac{{\triangle E}_{S}}{{\triangle E}_{\tau }}\right)}^{2}\left(\tau \,\ll\, {L}^{2}/D\right)$$

Therein the D value is estimated to range from 5.2 × 10^−12^ to ~10^−13^ cm^2^/s during discharge and range from 7.2 × 10^−12^ to ~10^−12^ cm^2^/s during charge (Fig. 7d). Although this diffusion performance is achieved under the solid-state architecture and conversion reaction mode, its D value is even high than those (1.9 × 10^−13^−5.7 × 10^−14^ cm^2^/s) of LiFePO_4_ and Li(Ni_0.5_Mn_0.5_)O_2_ insertion-type cathodes tested in combination with nonaqueous liquid electrolyte solutions^66,67^. Note that the D plots have the local minimums at ~2.5 V for discharge and at ~2.7 V for charge, i.e. at the beginning stages of conversion and reconversion reactions. At that time the nucleation or splitting of conversion products (e.g. LiF) with the formation or migration of multiphase interfaces would slow down the diffusion of Li across these phase boundaries. The D values are overall higher during charge than during discharge, in view of the more synchronous multiphase transformation and faster phase boundary healing during charge^1^. Figure 7e and Supplementary Table 10 show the evolution of the EIS measurements of Li|LiTFSI-PEO-0.2AlF_3_|FeF_3_ cell at different cycling stages. The interface resistance can be seen from the size of depressed or merged semicircles in the Nyquist plots, which contain the contributions of SEI and charge transfer resistances^68^. After 10 cycles, the interface resistance decreases from 89 to 35.8 Ω cm^2^. The interface activation is likely caused by the formation of more ionic conductive SEI as well as the more intimate contact between the fluorinated cathode and polymeric solid electrolyte. Subsequently, the semicircle size further shrinks and gradually reaches a minimum of 30.7 Ω cm^2^ after 250 cycles. The interface resistance can still be controlled at 40.6 Ω cm^2^ even after long 350 cycles. The stabilization of interface resistance is responsible for the durable cycling of all-solid-state Li||FeF_3_ battery system even under the pouch-cell configuration and at the temperature of 30 °C.

In order to estimate the promotion effect of electrolyte fluorination on the reversibility of conversion reaction in solid-state Li||FeF_3_ battery, we carried out ex situ XPS measurements on the positive electrodes disassembled from Li|LiTFSI-PEO|FeF_3_ and Li|LiTFSI-PEO-0.2AlF_3_|FeF_3_ coin cells in the terminal charging state after undergoing 10 cycles at 358 mA/g at 60 °C. Figure 7f–h shows the corresponding Fe 2*p*, Li 1*s*/Fe 3*p* and F 1*s* spectra for both the cycled cathodes. The peaks in Fe 2*p*_3/2_ can be roughly deconvoluted into Fe^3+^–F at 714.5 eV and Li–Fe–F/Fe^2+^–F around 710.2 eV^69^. The AlF_3_ reinforcement enables the higher fraction of Fe^3+^–F component, i.e. the better reconversion reversibility. Otherwise, the potential F loss triggers the more accumulation of F-deficient species and formation of metallic Fe^0^ at 706.2 eV even at the charge state in the case of LiTFSI-PEO system^70^. The Fe species without sufficient fluorination activation can irreversibly evolve into “dead Fe” (i.e., electronically disconnected iron metal particles) residual during long-term cycling, which is responsible for the fast capacity degradation of FeF_3_ cathode^71^. This phenomenon is in accordance with the higher intensities of LiF and Li–Fe–F peaks at 55.9 and 55 eV respectively in Li 1*s* of LiTFSI-PEO system^72^. Therein the Fe 3*p* peaks of Fe^0^ and Li–Fe–F also correspondingly appear at 52.7 and 57.8 eV respectively^13,69^. The accumulation of LiF component with higher concentration is companied with the residual of “dead Fe” in LiTFSI-PEO system. Therefore the passivation effect at cathode side is more serious in the AlF_3_-free cells. In view of the intimate contact between AlF_3_-dissoluble electrolyte and cycled FeF_3_ cathode during repeated lithiation/delithiation processes, the migration of F-ion from AlF_3_ to cathode likely occurs, leading to the formation of weak-bonded F signal at 682.1 eV in F 1*s*^73^. Such a low binding energy peak also appears in some F-ion conductors with weak-bonded F^74^. These “semi-free” F-ions can compensate for the F loss from cycled FeF_3_ and keep the sufficient fluorination environment, which is favorable for the retention of high capacity during cycling. The higher F concentration in LiTFSI-PEO-0.2AlF_3_-suported cathode is also indicated from the more intensive Fe–F peak at 684.8 eV^13^. Note that in C 1*s* spectra the fraction of C-C (at 284.8 eV) is higher than that of C–O–C (at 286.6 eV) for LiTFSI-PEO-0.2AlF_3_ system^53^, and the fraction tendency is opposite for LiTFSI-PEO system (Supplementary Fig. 30). This discrepancy indicates that the HS-AlF_3_ pillaring can suppress the extrusion of PEO segments (C–O–C) into cathode and the deformation of C-C electron wiring network. The optimized distribution of conductive carbon is responsible for the appearance of carbide peak (e.g. Li_x_C, at 282.2 eV) as a consequence of favorable lithiation of C^52^. These XPS results further reveal that the fluorinated polymer electrolyte can optimize the reversibility of conversion reaction at the cathode interface, thus improving the cycling stability and rate capability even under the all-solid-state architecture.

## Discussion

In summary, we proposed a strategy of dual fluorination on conversion-type cathode and polymer electrolyte to develop thin-lamination all-solid-state Li metal batteries with high capacity and durability. The PEO-based electrolyte is fluorinated by tailored mesoporous AlF_3_ self-assembled nanoparticles with strong Lewis acidity. The strong interaction between high-specific-surface AlF_3_ and PEO chains enables the formation of corrugated morphology and the improvement of ionic conductivity of composite electrolyte. The Raman spectra confirm the promotion effect of AlF_3_ on LiTFSI salt dissociation and the first-principles calculation indicates the strong adsorption effect of AlF_3_ surface towards TFSI^−^ anions, leading to a high Li-ion transference number of 0.67 for this fluorinated solid electrolyte. The AlF_3_ pillared polymer can trigger the enrichment of more conductive Li_2_O as dominant SEI component on the cycled Li metal anode and mitigate the interfacial passivation by attenuating the Li_2_CO_3_ component. The optimized Li symmetrical cells show quite small interfacial resistance and overpotential during long-term aging and cycling processes (e.g. 25, 50, and 75 mV at 0.1, 0.2, and 0.4 mA/cm^2^, respectively). The fluorinated interface of solid electrolyte can provide the extra F resource to compensate for the F loss from fluoride cathode during conversion reaction and enables the better reconversion reversibility. The corresponding all-solid-state Li||FeF_3_ batteries can achieve a reversible discharge capacity as high as ~600 mAh/g based on pouch-type cell configuration (at 153 mA/g) with thin electrolyte membrane (45 μm), as well as the long cycling stability (at least 900 cycles at 700 mA/g with discharge capacity retention of ~200 mAh/g) and high-rate performance (260 mAh/g at 3.5 A/g) based on coin cell configuration at 60 °C.

## Methods

### Material preparation

HS-AlF_3_ was synthesized via a sol-gel process. Typically, 18.76 g of Al (NO)_3_•9H_2_O (≥99%, Shanghai Aladdin Bio-Chem Technology Co., Ltd.) was first added to 50 mL of ethylene glycol (≥99.5%, GC, Shanghai Aladdin Bio-Chem Technology Co., Ltd.) to control the Al^3+^ concentration in 1 mol/L. The mixture was kept for stirring (at the speed of 500 rmp) at 50 °C until it turned out to be a transparent and uniform solution. Then, 6.7 mL of hydrofluoric acid (HF, 40 wt%, Sinopharm Chemical Reagent Co. Ltd.) was added to the solution followed by stirring (at the speed of 500 rmp) for more 6 h. The transparent solution was aged at 90 °C for 24 h and then at 120 °C for 24 h. A dry gel with light-yellow color was obtained by the transformation from the corresponding wet gel. Then the dry gel was placed in an open crucible and calcinated in a muffle furnace (containing air) at 400 °C for 4 h with a heating rate of 2 °C/min. Finally, a light brown powder was obtained.

### Fabrication of LiTFSI-PEO-AlF_3_ solid electrolytes

The composite solid polymer membrane was fabricated by a solution-casting process. Firstly, 360 mg of LiTFSI (99.95%, Sigma-Aldrich) and 880 mg of PEO (≥99% with an average Mv of about 2,000,000, Shanghai Aladdin Bio-Chem Technology Co., Ltd.) powder (with a ratio of [EO]:Li^+^ equal to 16:1) was added to 20 mL of acetonitrile. Then the HS-AlF_3_ powder with different weight ratio (0%, 5%, 10%, 20% or 30%) was added to the solution. The mixture was stirred (at the speed of 500 rmp) at 30 °C for at least 12 h and a reddish-brown solution was obtained. Then the solution was casted on a PTFE plate (with 10 cm in diameter and 3 mm in thickness) and evaporated at 25 ± 3 °C. At last, the electrolyte membrane was peeled off from the PTFE mold and dried for 12 h in vacuum at 60 °C for further use.

### Preparation of the LiFePO_4_-, LiNi_0.8_Co_0.1_Mn_0.1_O_2_- and, FeF_3_-based positive electrodes

To prepare the positive electrodes used in the all-solid-state cell assembly and testing, LiTFSI-PEO was introduced to act as ionic conductor. LiFePO_4_ (with a purity of 98.52% and carbon coating of 1.48%, and with an average particle diameter D50 of 1.3 μm) and NCM811 (without carbon coating, with a diameter D50 value of 3.8 μm) were commercially obtained from Hefei Ke Jing Materials Technology Co., Ltd. and FeF_3_ was synthesized by an ionic liquid based ionothermal fluorination method^75^. Typically, the dehydrated FeF_3_ with a hexagonal tungsten bronze (HTB) structure was prepared in 1-decyl-3-methylimidazolium tetrafluoroborate (C_10_mimBF_4_, Aldrich, >98%). Firstly, 1 g of FeCl_3_∙6H_2_O powder (99.99%, Aldrich) precursor was added to 10 mL C_10_mimBF_4_. The mixture was then stirred (at 500 rpm) at 50 °C for 6 h to form a homogeneous solution. Next, 5 wt% of single-walled carbon nanotubes (SWNTs) were added to the C_10_mimBF_4_ solution with further stirring (at 500 rpm) at 50 °C for more 12 h to form a black solution, where SWNTs were well dispersed. Afterward, the black solution was continuously heated and stirred (at 500 rpm) at 230 °C for 12 h. The obtained black precipitated product was then washed with acetone at 8000 rpm and centrifuged for 5 times to remove the residual ionic liquid and other impurities, and subsequently dried under vacuum at 80 °C for 20 h, to obtain the final product. In the positive electrode preparation process, the active material and conductive carbon black (Super P, Hefei Ke Jing Materials Technology Co., Ltd., >99.99%, with an average particle size of 35 ± 3 nm), PEO, and LiTFSI salt were manually mixed with a mortar in a weight ratio of 60:12:20:8 and then added to 12 mL of acetonitrile (≥99.5%, GC, Shanghai Aladdin Bio-Chem Technology Co., Ltd.) to form a uniform mixture. The mixture was stirred at 25 ± 3 °C for 12 h and then cast on a carbon-coated Al foil (Hefei Ke Jing Materials Technology Co., Ltd., ≥99.5%, 18 μm). The mass loading of LiFePO_4_ active material is about 2.02 or 10.2 mg/cm^2^, the loading of NCM811 is about 1.2 mg/cm^2^ and the loading of FeF_3_ is about 1, 3, or 4.2 mg/cm^2^.

### Ionic conductivity measurement

The two-electrode Swagelok-type SS|LiTSFI-PEO-AlF_3_|SS cells (where SS was commercially obtained from Hefei Ke Jing Materials Technology Co., Ltd., with a thickness of 3 mm) were used to measure the ionic conductivity by a Solartron frequency analyzer (1296–1260) in a frequency range from 10 MHz to 0.1 Hz with 10 data points per decade, under an open-circuit voltage in the potentiostatic mode, using an alternating current perturbation of 10 mV. The solid polymer membrane was shaped into pieces with a diameter of 10 mm and two pieces of ion-blocking stainless steel electrodes were placed on both sides of the electrolyte membrane. For the temperature-dependent measurement, the cells were placed into an oven (Shanghai Yiheng Technology Instrument Co., Ltd., with a temperature measurement error of 0.1 °C) and the impedance test was conducted from 70 to 30 °C with a temperature step of 10 °C. The cells were kept at each temperature point for an hour to fully reach a stable state. The ionic conductivity (ρ) of solid polymer electrolyte is calculated from the following equation:

4 $$\rho=\frac{L}{{RS}}$$

where *R* is the resistance value (i.e. the semicircle size or the abscissa intercept on the impedance spectra), L is the thickness of membrane (measured by a high-precision digital display micrometer) and S is the contacting area of electrode. The Li-ion transference number *t*_Li+_ of polymer electrolyte was calculated by a chronoamperometry method. A polarization of 10 mV was applied to Li|PEO-LiTFSI-AlF_3_|Li coin cell (with a Li foil thickness of 450 μm and an electrolyte membrane thickness of 45 μm, and their diameters of 10 and 19 μm respectively) to obtain the corresponding current-time curve. The AC impedance spectra of Li|PEO-LiTFSI-AlF_3_|Li cells were measured at open-circuit voltage in the potentiostatic mode under an amplitude of 10 mV with the frequencies from 1 MHz to 10 Hz and with 10 data points per decade before and after polarization respectively.

### Interfacial resistance measurement

In order to obtain the interface resistance between lithium metal (450 μm in thickness and 10 mm in diameter) and polymer electrolyte LiTFSI-PEO-0.2AlF_3_ (45 μm in thickness and 19 mm in diameter) (or LiTFSI-PEO, 120 μm in thickness and 19 mm in diameter), we used the 2025-coin-type Li|polymer|Li cells for testing. The electrochemical impedance spectra (EIS) were obtained with a voltage bias of 10 mV in a frequency range from 10 MHz to 0.1 Hz with 10 data points per decade at 60 °C, under an open-circuit voltage in the potentiostatic mode. The cell was left at the open-circuit voltage for 1 h before the EIS measurement. In order to obtain the evolution of interface resistance over aging time, EIS measurement was performed every 24 h. The solid-state battery of Li|PEO-LiTFSI-0.2AlF_3_|FeF_3_ was also tested by EIS at the terminal discharge state to detect the evolution of interface resistance at different cycling stages. The areal-specific resistance value is calculated by dividing the measured interface resistance (from the size of depressed or merged semicircles in the Nyquist plots) by the effective contact area of the electrode sheet.

### Electrochemical energy storage measurements

The battery cycling performance of Li||LiFePO_4_, Li||FeF_3_, and Li||NCM811 coin cells based on LiTFSI-PEO-0.2AlF_3_ and LiTFSI-PEO polymer electrolytes were tested at 60 °C at different specific currents. In this testing, the positive electrode sheet was cut into the sheets with a size of 7 × 7 mm^2^, the electrolyte membrane is 45 μm in thickness and 19 mm in diameter, and the Li foil is 450 μm in thickness and 10 mm in diameter. The galvanostatic measurement was conducted by a Land battery tester (CT2001A Wuhan Land Electronic Co., Ltd.). The voltage ranges are 2.0–3.8 V for Li||LiFePO_4_ cells, 1.2–3.9 V for Li||FeF_3_ cells, and 3.0–4.2 V for Li||NCM811 cells. The electrochemical behavior of lithium plating/stripping was tested based on Li|PEO-LiTFSI-0.2AlF_3_|Li and Li|LiTFSI-PEO|Li symmetric cells under various specific currents. We used an electrochemical workstation (VersaSTAT3, AMETEK Scientific Instruments) to conduct the electrochemical floating measurement based on a Li|LiTFSI-PEO-0.2AlF_3_|NCM811 cell to estimate the electrochemical stability window, where the gradually increasing voltage was maintained for 10 h in each step and the current response was recorded accordingly. CV measurement was carried out based on the asymmetric cell of Li|PEO-LiTFSI-0.2AlF_3_|SS or Li|LiTFSI-PEO|SS and the voltage range is set from −0.5 to 5.5 V at a scanning rate of 0.5 mV/s. For the kinetic analysis of FeF_3_-based positive electrode in the framework of all-solid-state batteries, the Li|polymer|FeF_3_ cell was measured at varied scanning rates from 0.02 mV/s to 1 mV/s to estimate the pseudocapacitive contribution. In order to obtain the diffusion coefficient of Li, the GITT measurement was conducted at 35 mA/g with an intermittent time of 1 h, followed by an open-circuit voltage relation time of 6 h.

The solid-state Li||FeF_3_ pouch cells were prepared and measured as follows: The thin lithium foil (≥99.5%) with a thickness of 45 μm was purchased from China Energy Lithium Co., Ltd (Tianjin). The prepared FeF_3_-based positive electrode (single side coated, similar to the preparation process as aforementioned in coin cells) was cut into the sheets with a size of 6 × 4 cm^2^, and the loading of active material was about 1 mg/cm^2^. The lithium foil was cut into the pieces with a slightly larger size of 6.2 × 4.2 cm^2^, and the composite polymer membrane of LiTFSI-PEO-0.2AlF_3_ was also cut into a larger size of 6.5 × 4.5 cm^2^ to fully separate the FeF_3_ cathode and lithium metal anode. These sheets are stacked to assemble the pouch cells, which were put in a splint mold designed in our own lab with a pressure of about 20 KPa and placed in an oven at 60 °C for 4 h before the battery testing. To ensure the reproducibility of date, three cells have been tested at 60 °C for a single electrochemical experiment.

### Materials characterizations

X-ray diffractometer (XRD, D8 Discover, Bruker) was used to determine the structure and phase assignment of as-prepared HS-AlF_3_ powder and polymer membrane. The XRD measurements were conducted in a 2θ range of 10–80° at a scanning rate of 1.0°/min using Cu Kα radiation. Transmission electron microscopy (TEM, JEOL JSM-6700F, operated at 200 kV) and scanning electron microscopy (SEM, Magellan 400 L, FEI) with energy dispersive spectra (EDS) mapping were used for observation of morphology, texture and thickness of HS-AlF_3_ powder and polymer membranes. The surface morphology of cycled Li metal anode was also detected by SEM imaging after disassembling the Li|PEO-LiTFSI-0.2AlF_3_|Li symmetrical cell after cycling for 120 h. X-ray photoelectron spectroscopy (XPS, ESCAlab-250) with an Al anode source was used to detect the surface components of AlF_3_ particles, cycled lithium metal anodes and cycled FeF_3_ cathodes. For the ex situ XPS characterization and ex situ SEM measurement, the corresponding cells were carefully disassembled in an argon-filled glove box, and the cycled Li foil electrodes (at the plating stage) were peeled off from the electrolyte membranes and the cycled FeF_3_ positive electrode membranes (in the charged state) were peeled off from the carbon-coated aluminum foils. The cycled electrode and electrolyte samples were dried and sealed in an argon-filled container, and then were quickly transferred to the measurement equipment. However, for the XPS characterization, the sample was transiently exposed to dry air (about 15 s) during transfer to the measurement chamber. Therefore the prior etching process for 10 s was preformed to remove the thin O-contamination layer and the fusion polymer layer on Li surface before detecting the true interface information. Fourier transform infrared (FTIR) spectra of various polymer electrolytes of PEO-LiTFSI-0.2AlF_3_, LiTFSI-PEO and PEO were conducted by Bruker Tensor 27 in a wavelength range of 500–4000 cm^−1^. Raman spectra of PEO-LiTFSI-0.2AlF_3_ and LiTFSI-PEO were obtained on Renishaw invia in a frequency range from 100 to 3400 cm^−1^. Thermal gravimetric analysis (TGA) curves and differential scanning calorimeter (DSC) curves of polymer electrolyte samples were measured with TGA machine (Netzsch STA 409 PC) under a heating rate of 10 °C/min in a nitrogen atmosphere. The temperature range was set from the ambience temperature to 600 °C for TGA measurement and from −60 to 150 °C for DSC measurement. The Brunauer-Emmett-Teller (BET) method was used to estimate the specific surface area and pore diameter distribution of HS-AlF_3_ sample. The nitrogen sorption isotherms were obtained with an Autosorb-1 system (Quantachrome).

### DFT calculation

The first-principles calculations based on density functional theory (DFT) were employed to investigate the adsorption energy between AlF_3_ and TFSI^−^. Firstly, the (012) plane of AlF_3_ crystal structure with the supercell expansion of 3 × 3 × 1, TFSI and adsorption structure between AlF_3_ and TFSI were built, and their geometry optimization was performed by the CASTEP program. During the calculation process, the Perdew-Berke-Ernzerhof (PBE) method in the generalized gradient approximation (GGA) was adopted to describe the periodic boundary conditions and the inter-electronic exchange-correlation energy. The interaction potential between the ion core and valence electrons was achieved by the ultra-soft potential. The cut-off energy of plane wave was chosen as 500 eV in the wave vector K-space. And the Brillouin zone of 2 × 4 × 3 was chosen according to the special K-point of Monkhorst-Park. The calculation accuracy of the crystal structure system to reach the convergence state was set as follows: the total energy change of the system is within 10^−5 ^eV, the force acting on each atom in the unit cell is less than 0.02 eV/Å, the residual stress of unit cell and its tolerance deviation are within 0.05 GPa and 10^−3 ^Å, respectively. The free energies of AlF_3_ of (012) plane (*E*_AlF3_), TFSI (*E*_TFSI_) and adsorption structure of AlF_3_ and TFSI (*E*_AlF3_-_TFSI_) were obtained after geometry optimization. The adsorption energy between AlF_3_ and TFSI (*E*_adsorb_) was calculated according to the following formula: *E*_adsorb_ = *E*_AlF3_ + *E*_TFSI_ − *E*_AlF3-TFSI_. The same calculations methods were employed to investigate the adsorption energy between AlF_3_ and PEO. The free energies of AlF_3_ of (012) plane (*E*_AlF3_), PEO (E_PEO_) and adsorption structure of AlF_3_ and PEO (*E*_AlF3-PEO_) were obtained after geometry optimization. The adsorption energy between AlF_3_ and PEO (*E*_adsorb_′) was calculated according to the following formula: *E*_adsorb_′ = *E*_AlF3_ + *E*_PEO_ − *E*_AlF3-PEO_.

### Reporting summary

Further information on research design is available in the Nature Portfolio Reporting Summary linked to this article.

## Supplementary information

Supplementary Information

Reporting Summary

## Data Availability

All data generated or analyzed during this study are included in this published article and its supplementary information file. The source data that support the findings of this study are available from the corresponding author upon reasonable request.
