# Supplementary material for: Dual fluorination of polymer electrolyte and conversion-type cathode for high-capacity all-solid-state lithium metal batteries
Source: Nat Commun. 2022 Dec 23;13:7914. doi: 10.1038/s41467-022-35636-0 (PMC9789084; doi:10.1038/s41467-022-35636-0)
Supplement: Supplementary file 1 — Supplementary Information [file 41467_2022_35636_MOESM1_ESM.pdf]

Supplementary Information

Dual fluorination of polymer electrolyte and conversion-type cathode for high-capacity all-solid-state lithium metal batteries

Jiulin Hu [1,3], Chuanzhong Lai [1,2,3], Keyi Chen [1,3], Qingping Wu [1,3], Yuping Gu [1,2,3], Chenglong Wu [1,2,3] and Chilin Li [1,2,3] *

[1] State Key Laboratory of High Performance Ceramics and Superfine Microstructure, Shanghai Institute of Ceramics, Chinese Academy of Sciences, 585 He Shuo Road, Shanghai 201899, China.

Email: chilinli@mail.sic.ac.cn

[2] Center of Materials Science and Optoelectronics Engineering, University of Chinese Academy of Sciences, Beijing 100049, China.

[3] CAS Key Laboratory of Materials for Energy Conversion, Shanghai Institute of Ceramics, Chinese Academy of Sciences, Shanghai 201899, China.

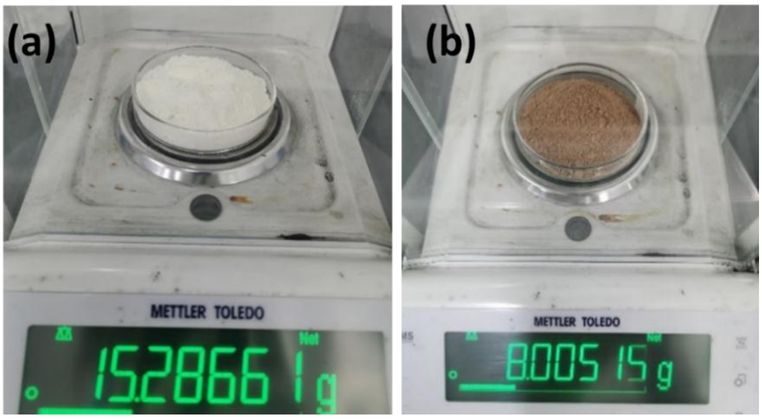

Supplementary Figure 1. Photos showing (a) 15.3 g of AlF_3 dry gel powder and (b) 8 g of final HS-AlF_3 powder. The weighing is carried out in air atmosphere.

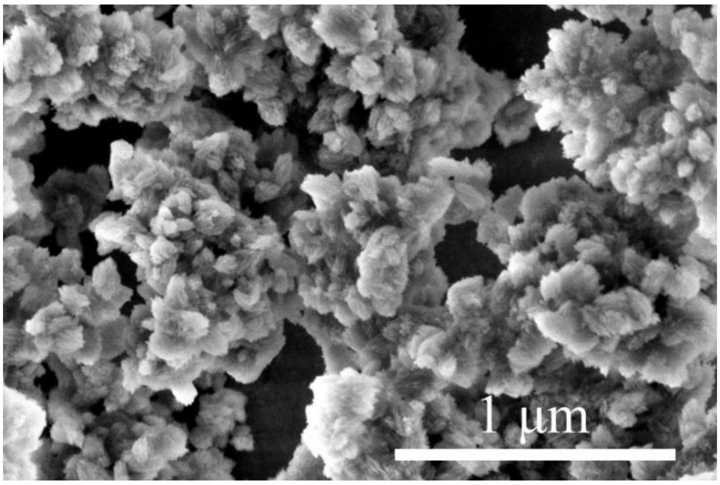

Supplementary Figure 2. SEM image of as-prepared HS-AlF_3 particles

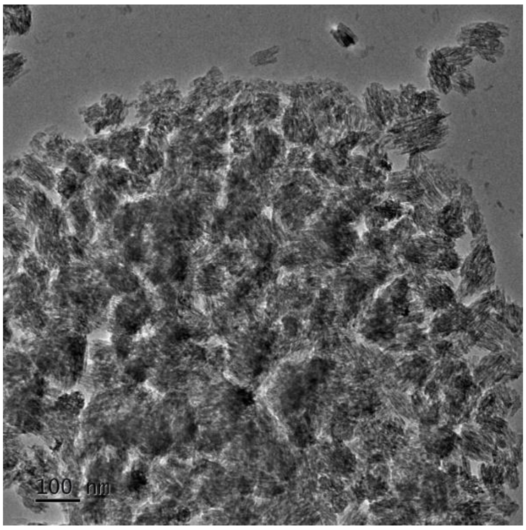

Supplementary Figure 3. TEM image of HS-AlF_3 particles

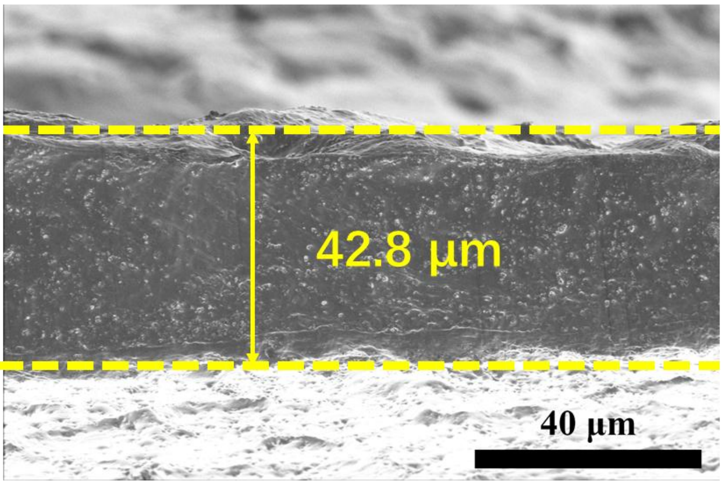

Supplementary Figure 4. SEM image of LiTFSI-PEO-0.2AlF_3 membrane, showing the cross-section morphology.

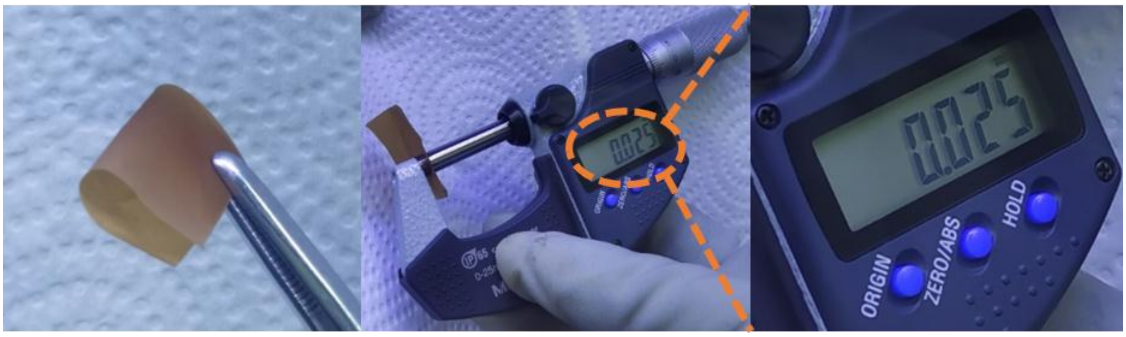

Supplementary Figure 5. HS-AlF_3 reinforced electrolyte membrane with a thinner thickness down to 25 μm. The thickness measurement is carried out in an Ar-filled glovebox by a high precision digital micrometer with the measurement error of 1 μm.

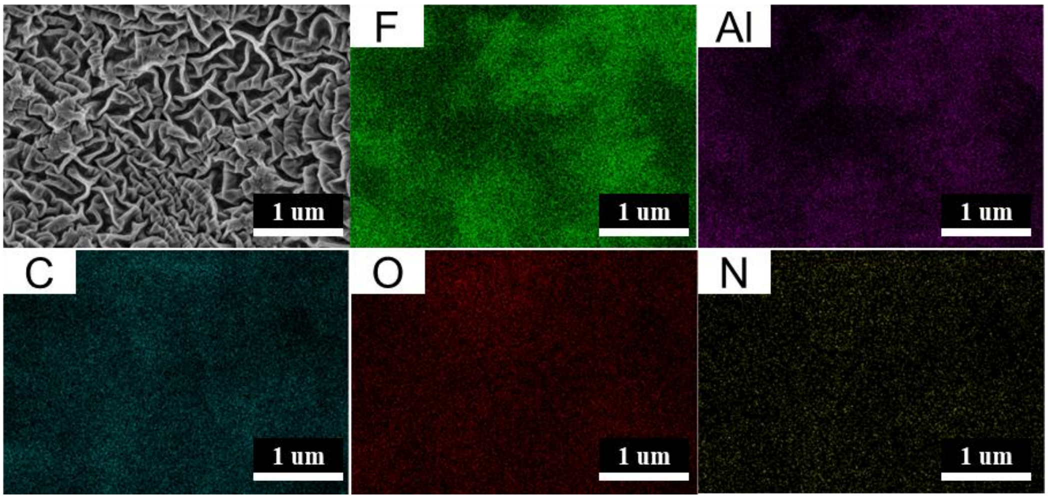

Supplementary Figure 6. SEM image and EDX element mapping of F, Al, C, O and N in the membrane of LiTFSI-PEO-0.2AlF_3 before cycling.

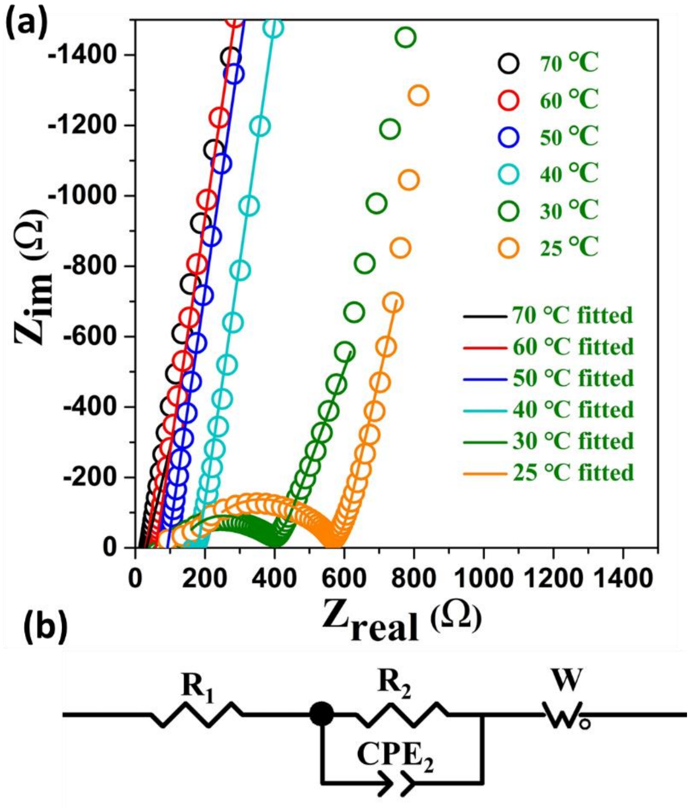

Supplementary Figure 7. (a) Nyquist plots of SS|LiTFSI-PEO-0.2AlF_3|SS cell and their fitting curves measured at different temperatures. (b) Equivalent circuit of these Nyquist plots. In this equivalent circuit, R_1 represents the ohmic resistance of cell, R_2 represents the ionic resistance of polymer membrane, CPE_2 represents the corresponding constant phase element, and W refers to the Warburg impedance describing both diffusion and accumulation of Li.

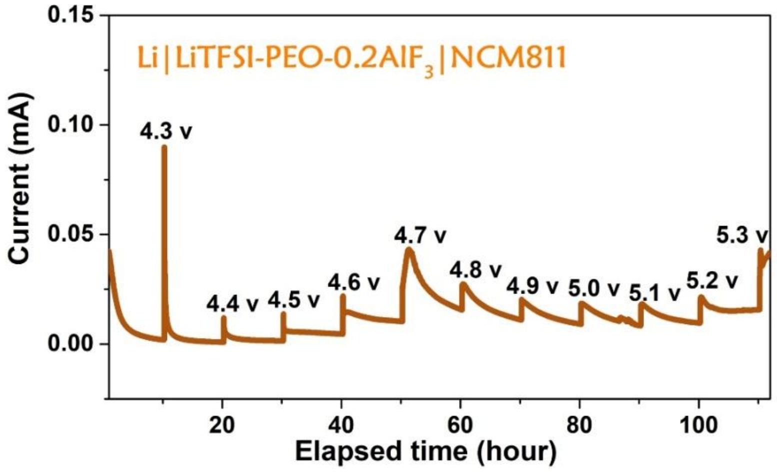

Supplementary Figure 8. Electrochemical floating experiment of Li|LiTFSI-PEO-0.2AlF_3|NCM811 coin cell at 60 ℃ to check the electrochemical window.

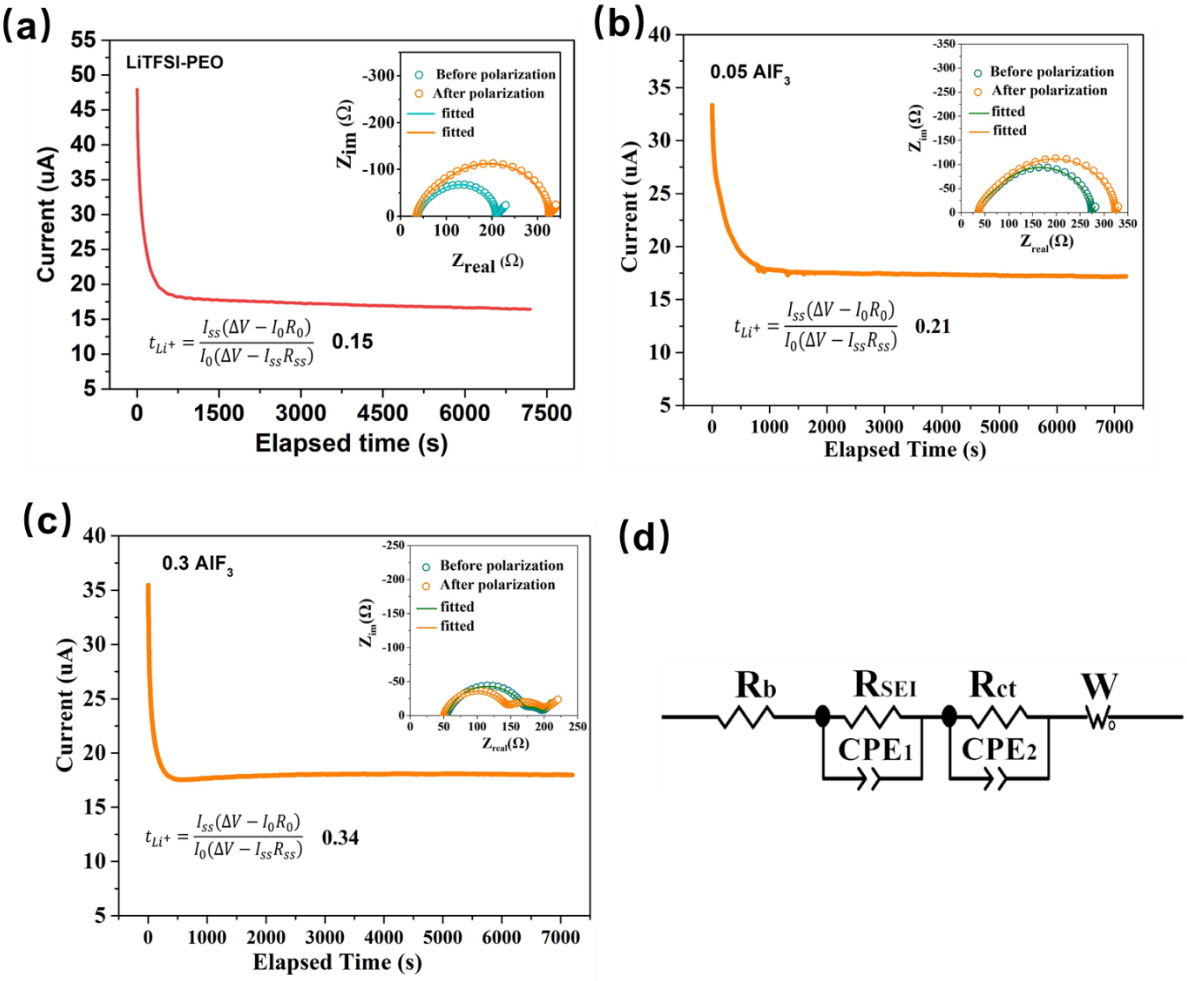

Supplementary Figure 9. Chronoamperometry curves of (a) Li|LiTFSI-PEO|Li, (b) Li|LiTFSI-PEO-0.05AlF_3|Li and (c) Li|LiTFSI-PEO-0.3AlF_3|Li coin cells at a voltage bias of 10 mV for a duration time more than 7000 s, insets: AC impedance spectra of corresponding symmetric cells before and after polarization at 60 ℃. (d) Equivalent circuit of these Nyquist plots. In this equivalent circuit, R_b represents the ohmic resistance of cell, R_{SEI} represents the lithium diffusion resistance in SEI, and R_{ct} presents the charge transfer resistance at the electrolyte-electrode interface. The CPE_1 and CPE_2 represent the corresponding constant phase elements. W refers to the Warburg impedance describing both diffusion and accumulation of Li.

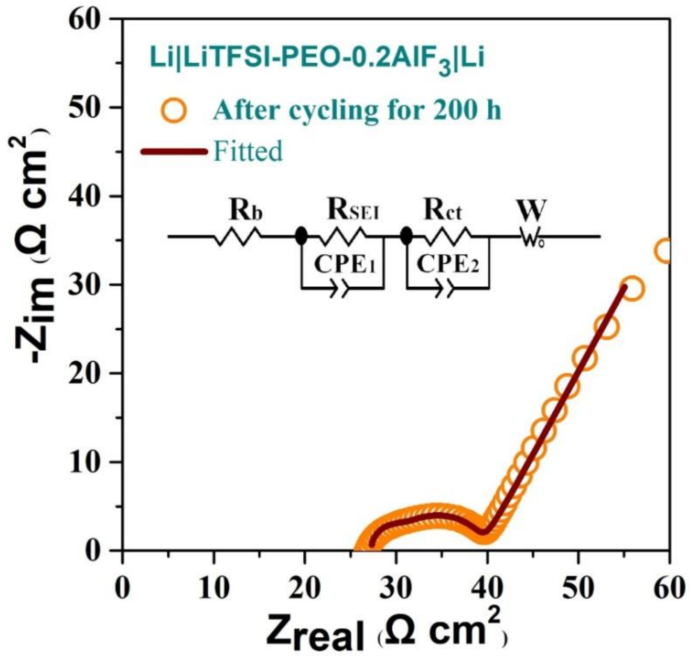

Supplementary Figure 10. AC impedance spectrum of Li|LiTFSI-PEO-0.2AlF_3|Li symmetric coin cells at 60 °C after cycling for 200 h at 0.1 mA/cm^2@ 0.1 mAh/cm^2. In the inset equivalent circuit, R_b represents the ohmic resistance of cell, R_{SEI} represents the lithium diffusion resistance in SEI, and R_{ct} presents the charge transfer resistance at the electrolyte-electrode interface. The CPE_1 and CPE_2 represent the corresponding constant phase elements. W refers to the Warburg impedance describing both diffusion and accumulation of Li.

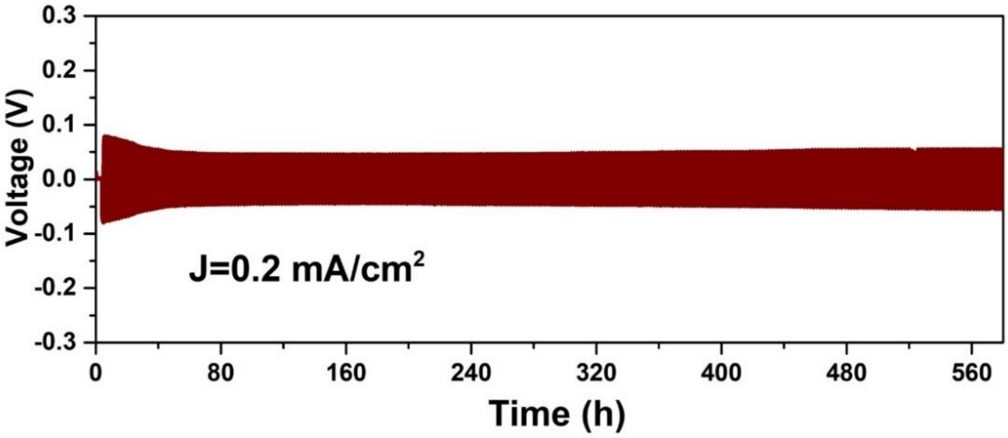

Supplementary Figure 11. Galvanostatic Li plating/stripping cycling performance of Li|LiTFSI-PEO-0.2AlF_3|Li symmetric coin cell at 0.2 mA/cm^2@0.2 mAh/cm^2 at 60 ℃.

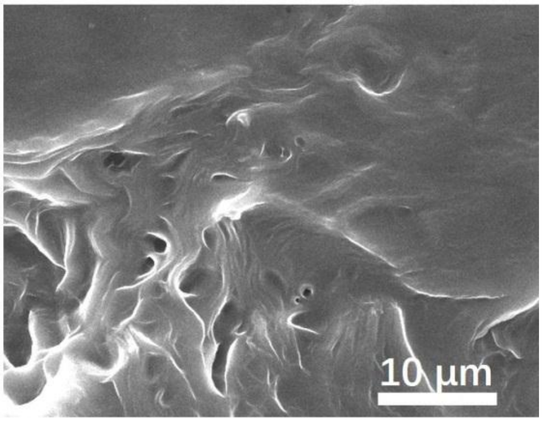

Supplementary Figure 12. Ex situ SEM image of surface morphology of uncycled LiTFSI-PEO.

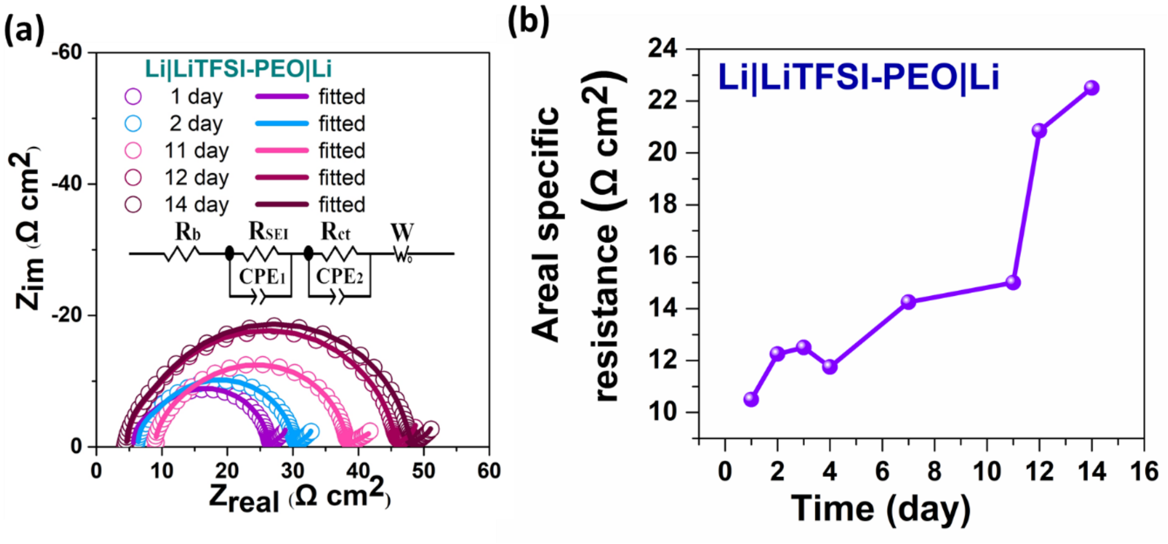

Supplementary Figure 13. (a) Electrochemical impedance spectra of Li|LiTFSI-PEO|Li symmetric coin cell under different aging times at 60 ℃. In the inset equivalent circuit, R_b represents the ohmic resistance of cell, R_{SEI} represents the lithium diffusion resistance in SEI, and R_{ct} presents the charge transfer resistance at the electrolyte-electrode interface. The CPE1 and CPE2 represent the corresponding constant phase elements. W refers to Warburg impedance describing both diffusion and accumulation of Li. (b) Areal specific resistance evolution as a function of aging time based on Li|LiTFSI-PEO|Li cell.

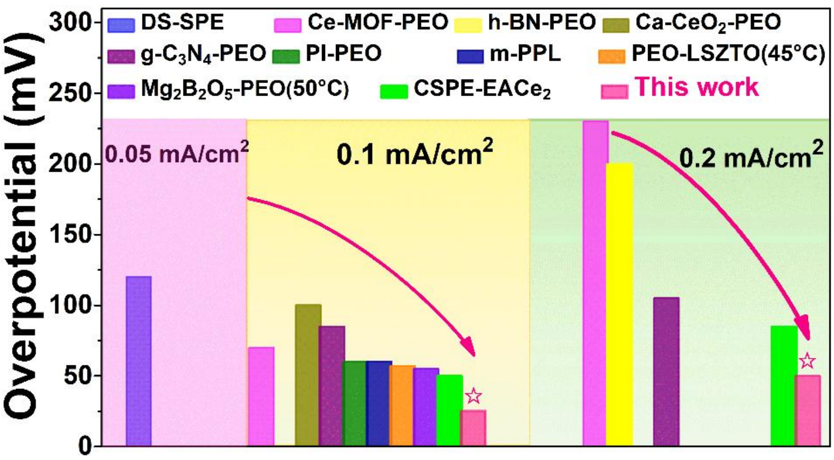

Supplementary Figure 14. Comparison of overpotentials of Li||Li symmetric cells based on LiTFSI-PEO-0.2AlF_3 electrolyte with other reported polymer-based electrolytes (based on g-C_3N_4-PEO[S1], Ce-MOF-PEO[S2], Ca-CeO2-PEO[S3], $Mg_2B_2O_5$-PEO(50 ℃)[S4], CSPE-$EACe_2$[S5], DS-SPE[S6], h-BN-PEO[S7], PI-PEO[S8], m-PPL[S9], PEO-LSZTO(45 ℃) [S10]) at different current densities.

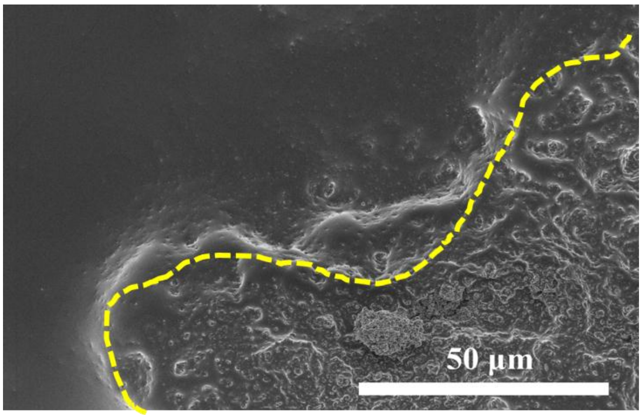

Supplementary Figure 15. Ex situ SEM image of surface of cycled Li anode (at the plating state) peeled from LiTFSI-PEO-0.2AlF_3 membrane after cycling of Li|LiTFSI-PEO-0.2AlF_3|Li symmetric cell for 300 h at 0.1 mA /cm^2 60 ℃.

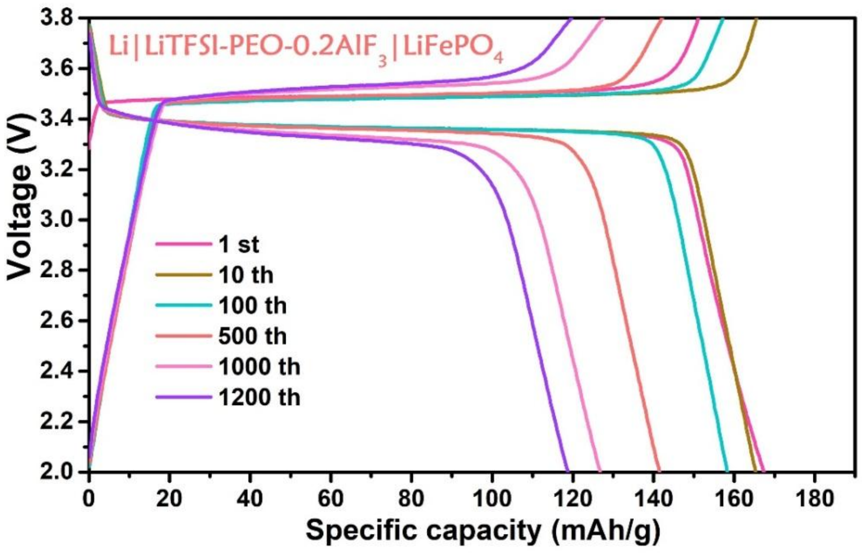

Supplementary Figure 16. Galvanostatic charge discharge curves of Li|LiTFSI-PEO-0.2AlF_3|LiFePO_4 coin cell at different cycling stages in a voltage range of 2-3.8 V at 60 ℃.

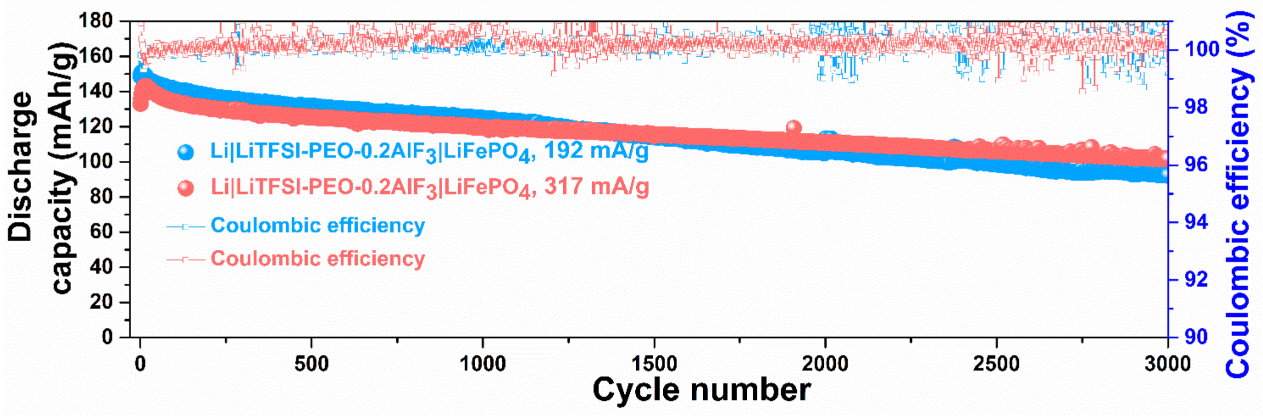

Supplementary Figure 17. Discharge capacities of Li|LiTFSI-PEO-0.2AlF_3|LiFePO_4 coin cells measured at 60 ℃ with a cathode loading of 1 mg/cm^2 as a function of cycle number at 192 and 317 mA/g.

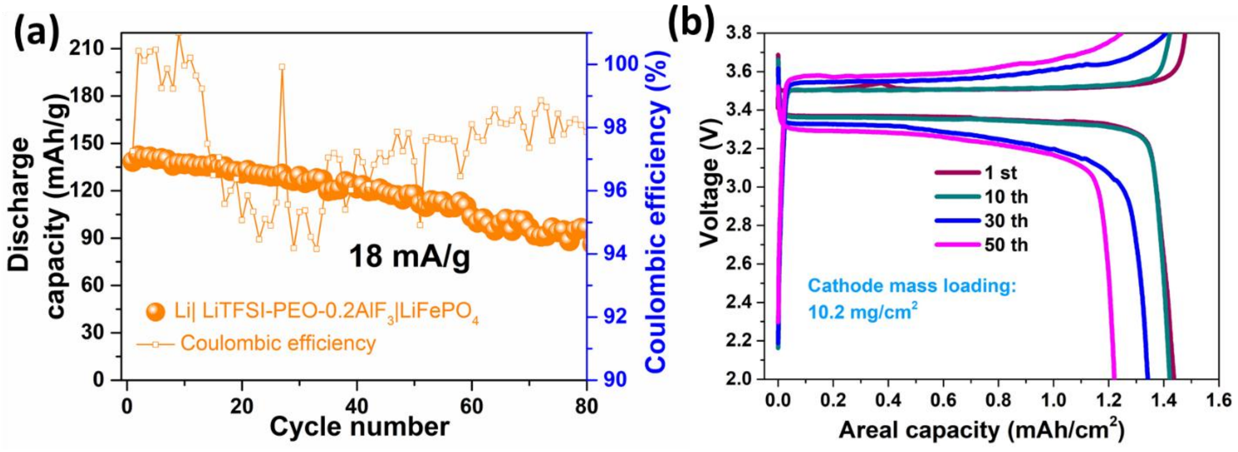

Supplementary Figure 18. (a) Discharge capacity and CE evolution of Li|LiTFSI-PEO-0.2AlF_3|$LiFePO_4$ coin cell based on high-loading cathode as a function of cycle number at 18 mA/g measured at 60 ℃. (b) Corresponding galvanostatic charge-discharge curves of Li|LiTFSI-PEO-0.2AlF_3| $LiFePO_4$ coin cell at different cycling stages in a voltage range of 2-3.8 V at 60 ℃.

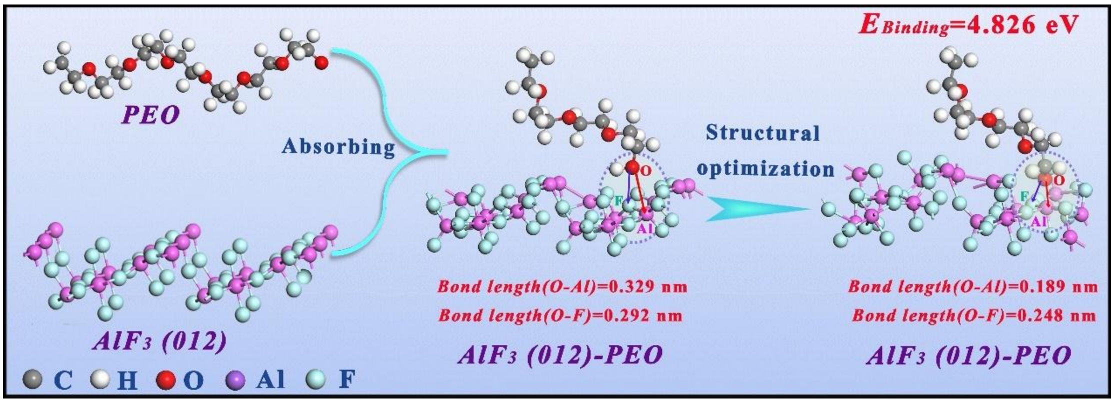

Supplementary Figure 19. Schematic illustration of the structures of PEO chain and AlF_3 (012) plane, as well as their interaction mode with the numerical values of the bond lengths and adsorption binding energy between AlF_3 and PEO terminated chain.

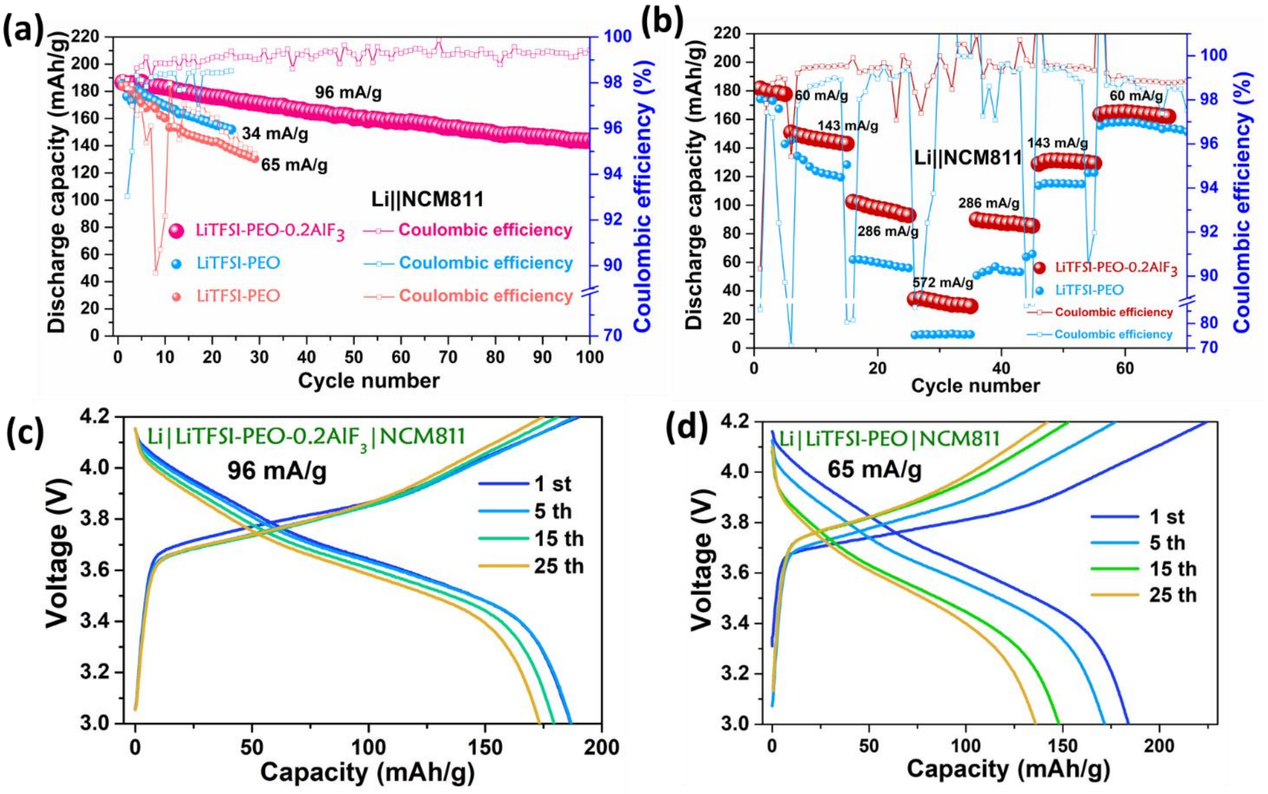

Supplementary Figure 20. Electrochemical cycling performance of all-solid-state polymer cells based on NCM811 cathodes measured at 60 ℃. (a) Comparison of discharge capacities based on Li|LiTFSI-PEO-0.2AlF_3|NCM811 coin cells at 96 mA/g and Li|LiTFSI-PEO|NCM811 coin cells at 34 and 65 mA/g as a function of cycle number. (b) Comparison of rate performances based on Li|LiTFSI-PEO-0.2AlF_3|NCM811 and Li|LiTFSI-PEO|NCM811coin cells at different rates from 60 to 572 mA/g. Galvanostatic charge–discharge curves of (c) Li|LiTFSI-PEO-0.2AlF_3|NCM811 at 96 mA/g and (d) Li|LiTFSI-PEO|NCM811 coin cells at 65 mA/g at different cycling stages in a voltage range of 3-4.2 V.

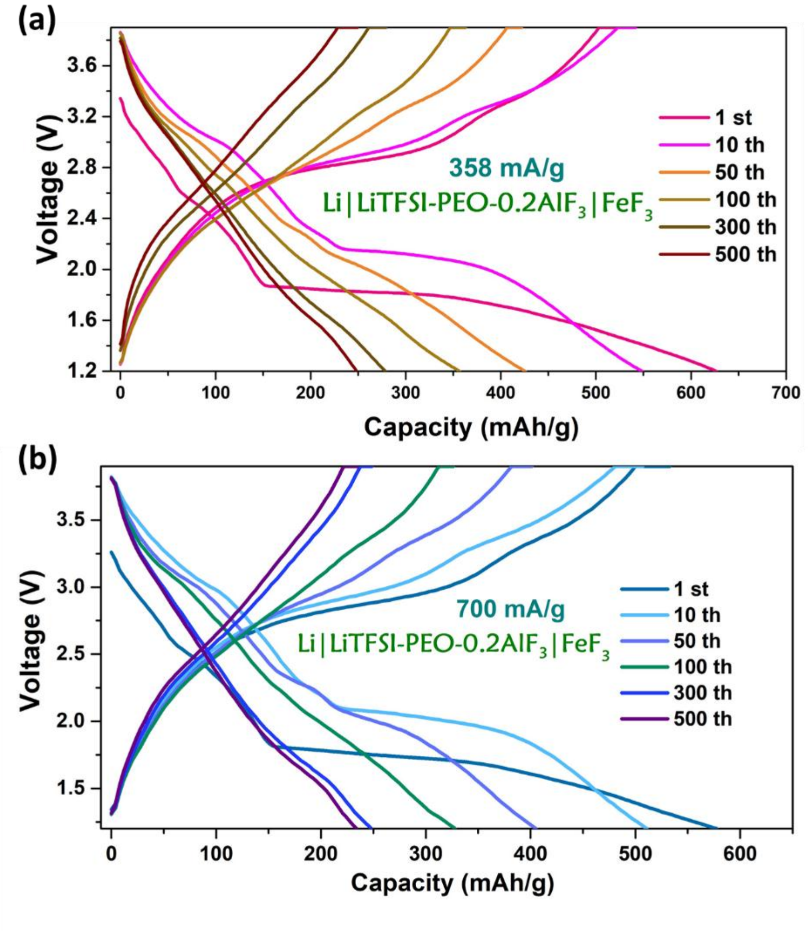

Supplementary Figure 21. Charge-discharge curves of Li|LiTFSI-PEO-0.2AlF$_3$|FeF$_3$ coin cells measured at 60 ℃ at different cycling stages in a voltage range of 1.2-3.9 V at (a) 358 and (b) 700 mA/g.

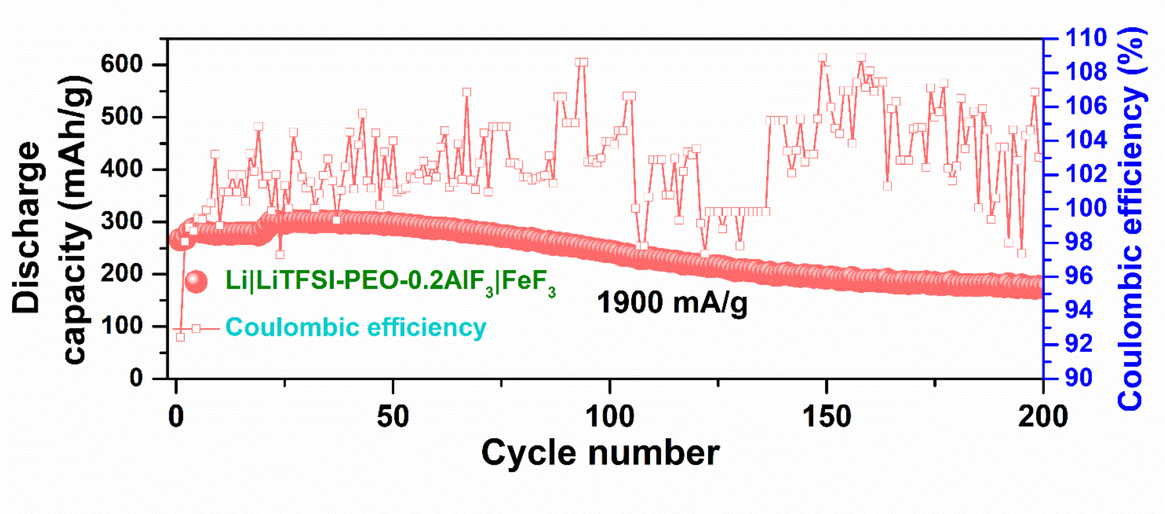

Supplementary Figure 22. Discharge capacities of Li|LiTFSI-PEO-0.2AlF$_3$|FeF$_3$ coin cell as a function of cycling number at 1900 mA/g and at 60 ℃.

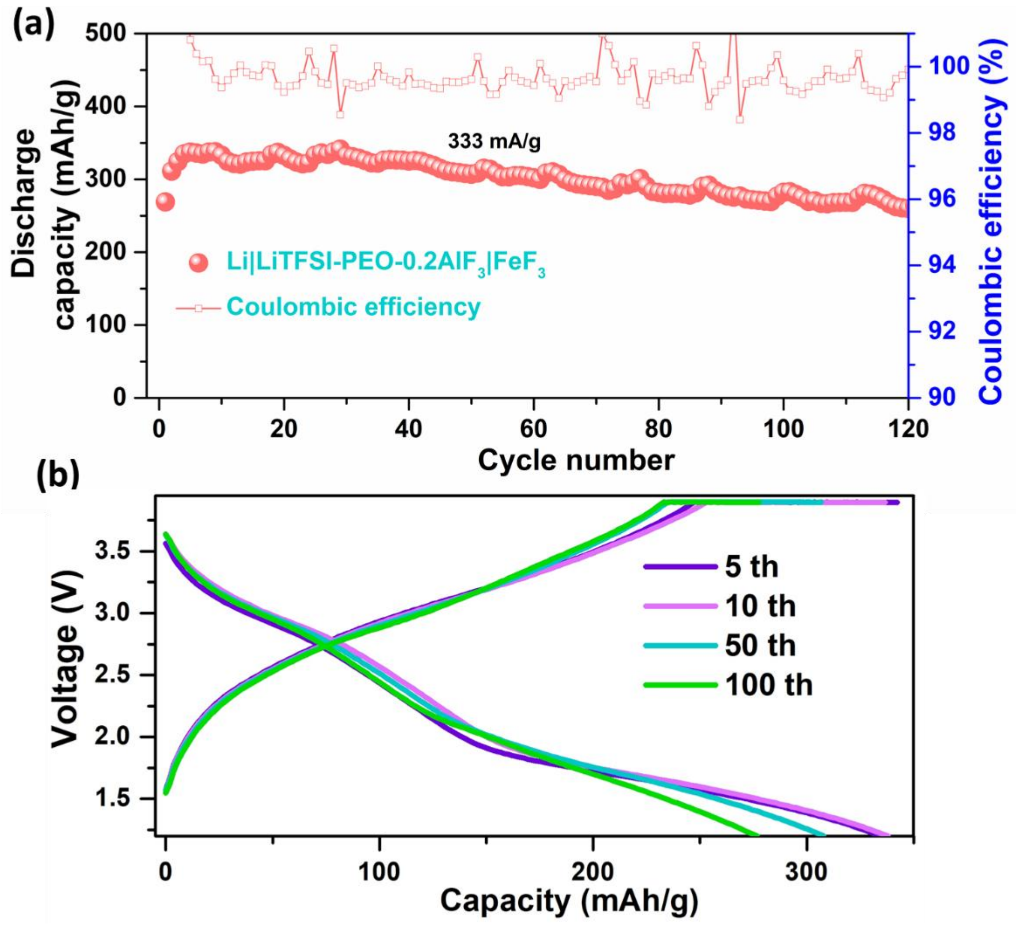

Supplementary Figure 23. Discharge capacities of Li|LiTFSI-PEO-0.2AlF_3|FeF_3 coin cell as a function of cycle number at 333 mA/g at 30℃. (b) Corresponding charge and discharge curves at different cycling stages in a voltage range of 1.2-3.9 V.

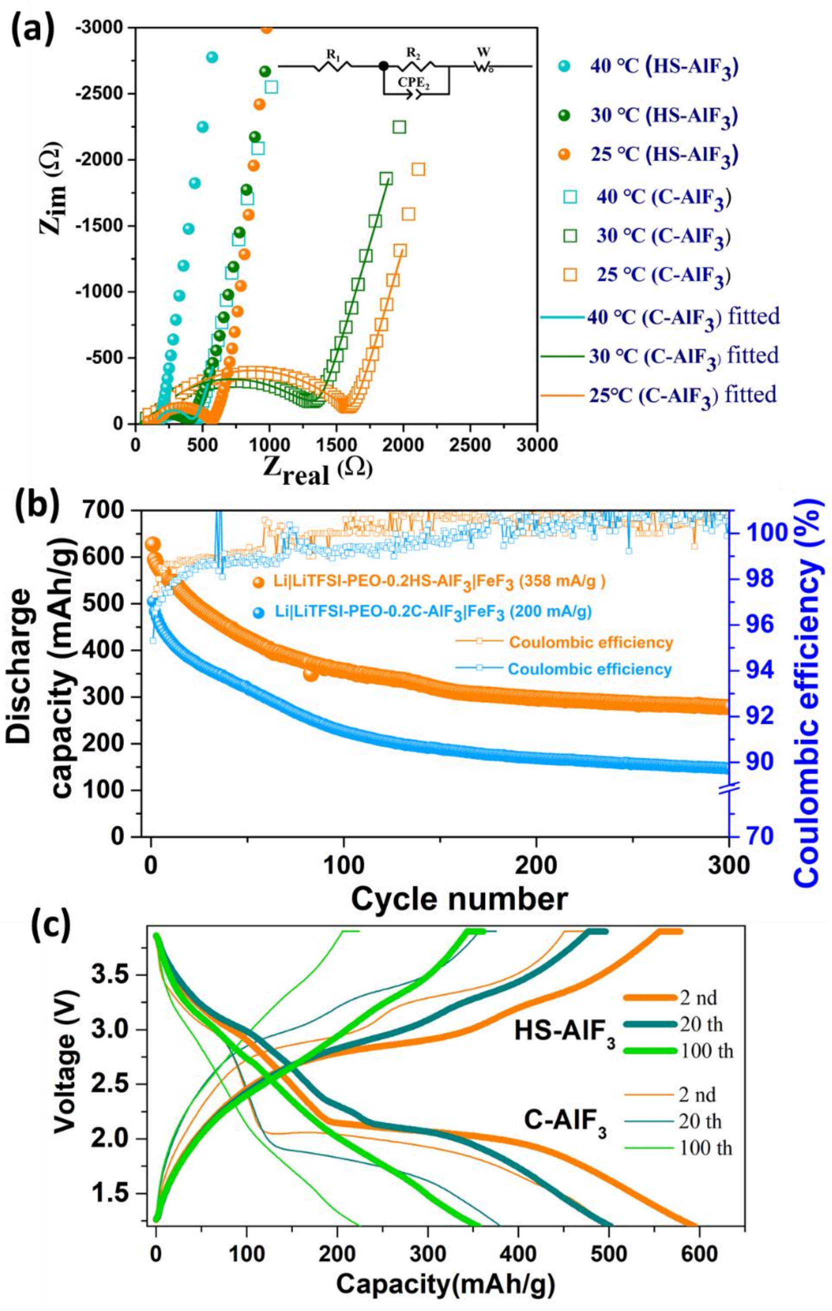

Supplementary Figure 24. (a) Nyquist plots of SS|LiTFSI-PEO-0.2C-AlF_3|SS and SS|LiTFSI-PEO-0.2HS-AlF_3|SS coin cells measured at different temperatures. In the inset equivalent circuit, R_1 represents the ohmic resistance of cell, R_2 represents the ionic resistance of polymer membrane, CPE_2 represents the corresponding constant phase element, and W refers to the Warburg impedance describing both diffusion and accumulation of Li. (b) Discharge capacity and CE evolution of Li|LiTFSI-PEO-0.2C-AlF_3|FeF_3 and Li|LiTFSI-PEO-0.2HS-AlF_3|FeF_3 coin cells as a function of cycle number at a specific capacity of 200 or 358 mA/g at 60 ℃. (c) Corresponding

charge and discharge curves at different cycling stages in a voltage range of 1.2-3.9 V at a specific capacity of 200 or 358 mA/g at 60 ℃.

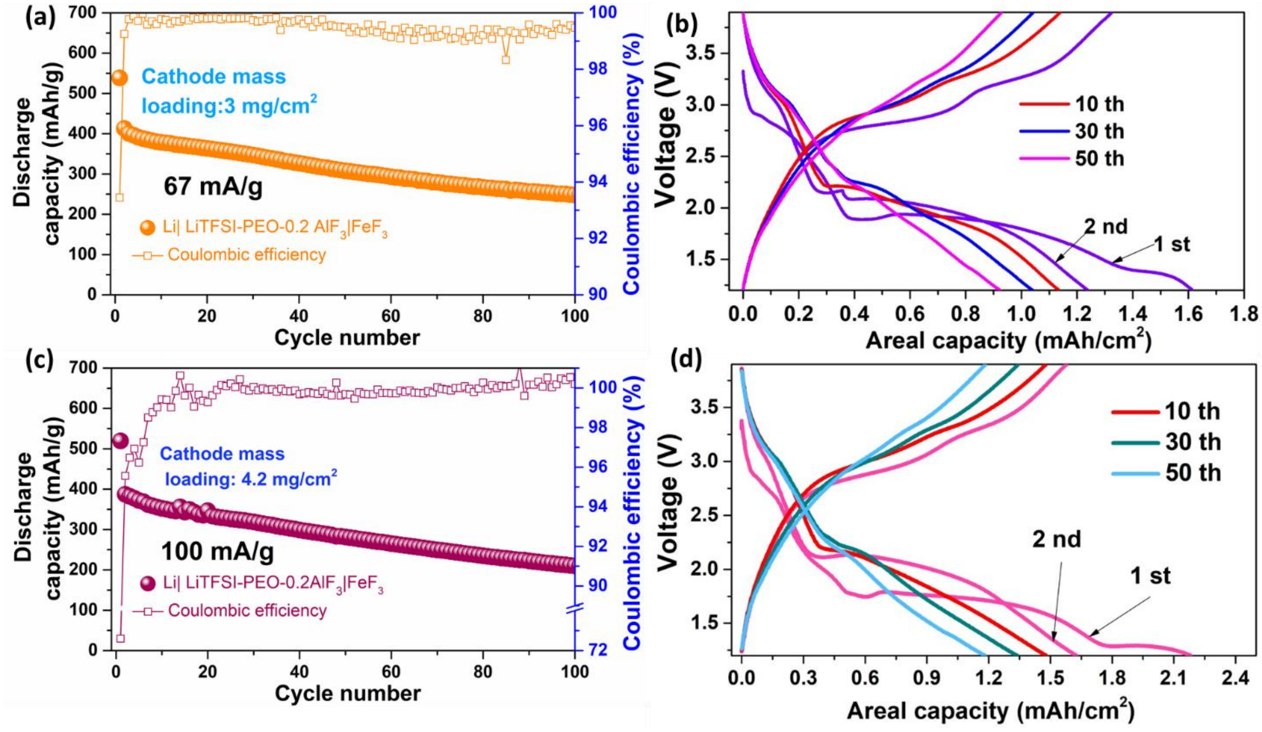

Supplementary Figure 25. Discharge capacity and CE evolution of Li|LiTFSI-PEO-0.2AlF_3|FeF_3 coin cells measured at 60℃ based on high-loading cathodes as a function of cycle number (a) at 67 mA/g and (b) 100 mA/g. Corresponding charge-discharge curves of Li||FeF_3 coin cells to display the areal capacity at different cycling stages (b) at 67 mA/g and (d) 100 mA/g.

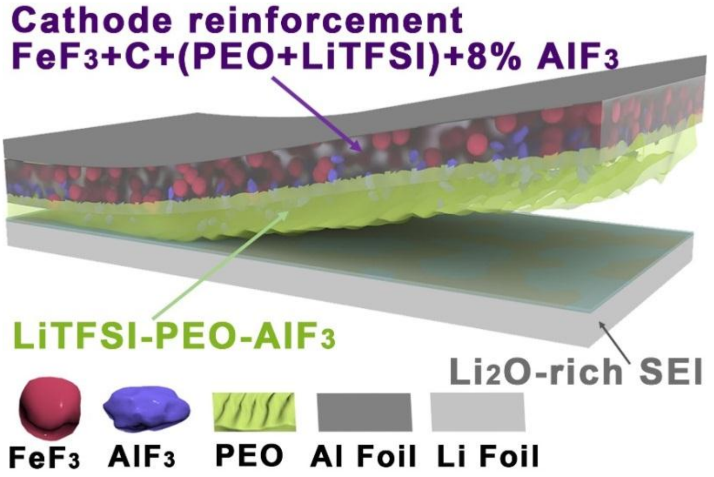

Supplementary Figure 26. Schematic configuration of Li|LiTFSI-PEO-AlF_3|FeF_3 cell with cathode reinforcement by the introduction of 8 wt% HS-AlF_3 and anode reinforcement by the enrichment of Li_2O in SEI.

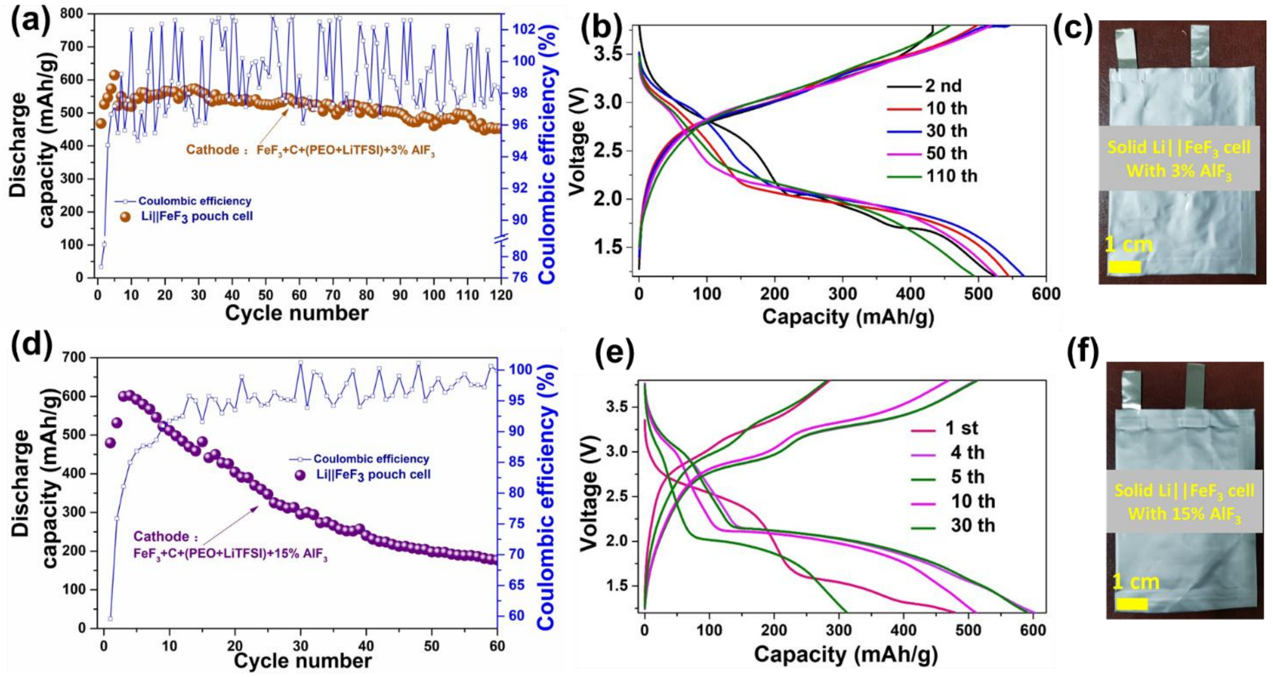

Supplementary Figure 27. (a) Cycling performance of pouch-type all-solid-state Li|LiTFSI-PEO-0.2AlF_3|FeF_3 cells measured at 60℃ with 3 wt% HS-AlF_3 addition at cathode side at 153 mA/g, (b) corresponding charge and discharge curves at different cycling stages, and (c) corresponding photographic picture of the pouch cell. (d) Cycling performance of pouch-type all-solid-state Li|LiTFSI-PEO-0.2AlF_3|FeF_3 cells measured at 60℃ with 15 wt% HS-AlF_3 addition at cathode side at 75 mA/g, (e) corresponding charge and discharge curves at different cycling stages, and (f) corresponding photographic picture of the pouch cell.

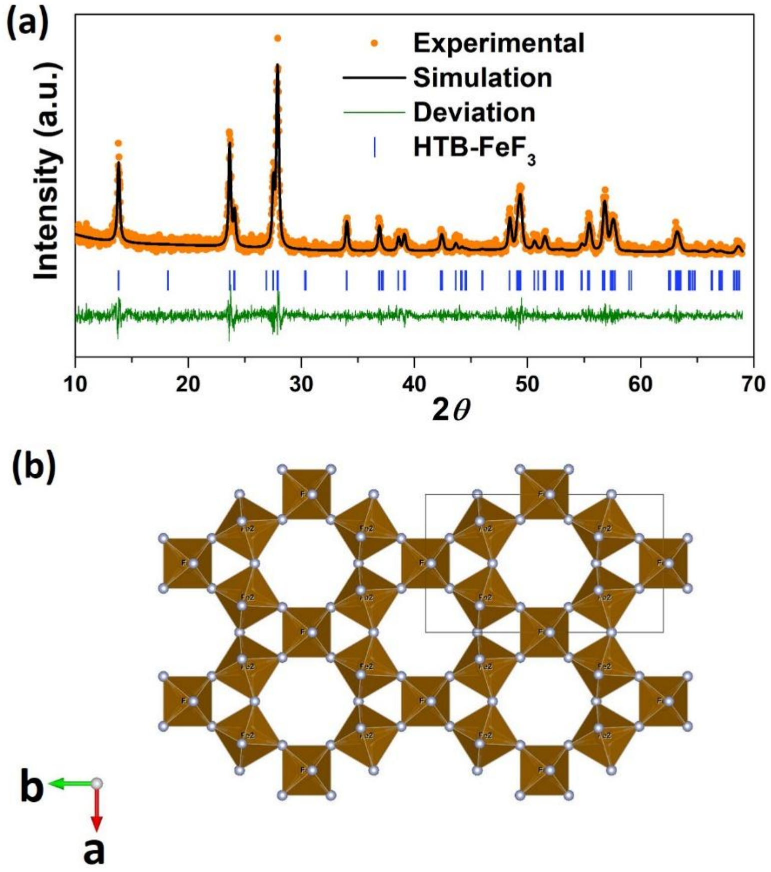

Supplementary Figure 28. (a) XRD pattern and corresponding Rietveld refinement of HTB-FeF_3. (b) Crystal structure of HTB-FeF_3 from the view of c axis, showing the typical feature of hexagonal tungsten bronze. The Rietveld refinement on the XRD pattern of FeF_3 results in a weighted residual factor (R_{wp}) of 26.075% and goodness of fit (GOF) of 1.10. The refinement result discloses the cell parameters of a = 7.394 Å, b = 12.762 Å, c = 7.514 Å with a cell volume of V_{cell} = 709.04 Å^3. Note that one cell contains 12 FeF_3 units, so the molar volume of FeF_3 can be calculated as: $V_m = N_A \times V_{cell}/12$, where N_A is the Avogadro constant (6.022×10^{23}/mol). The calculation V_m value is 3.56×10^{-5} m^3/mol.

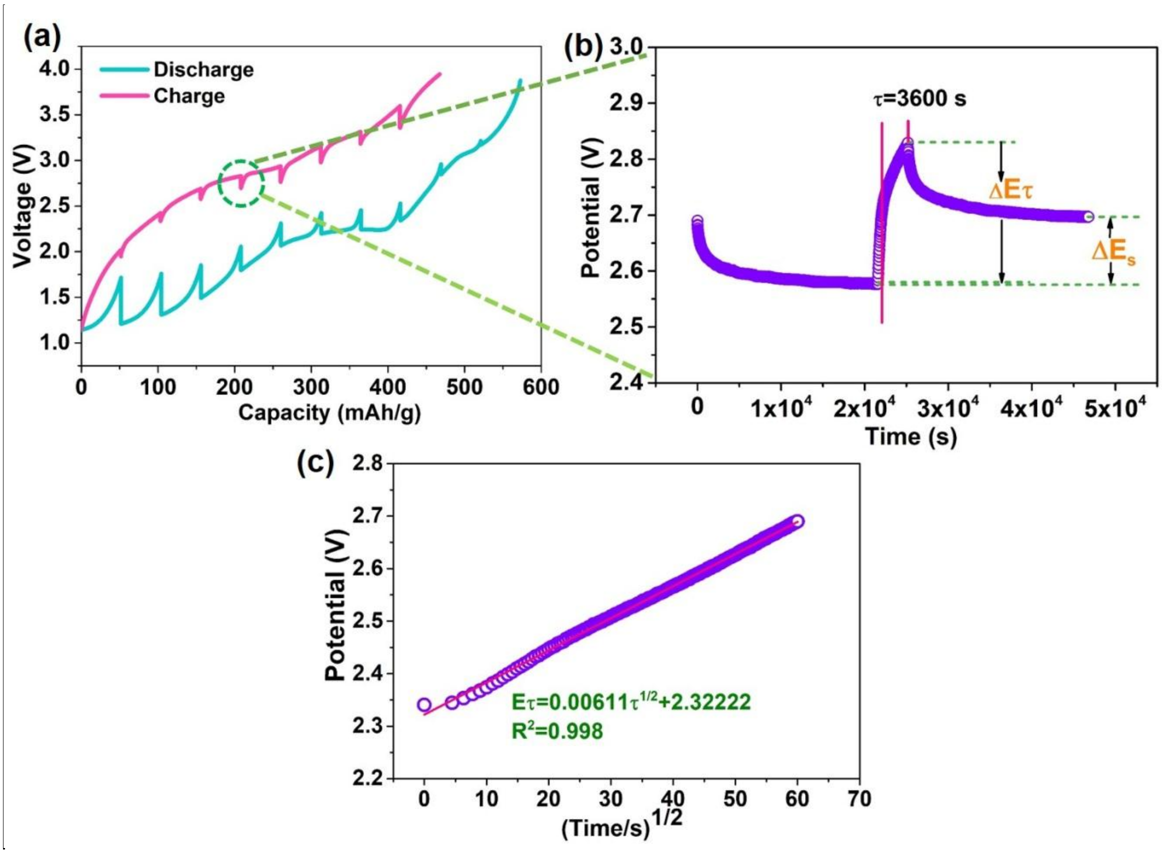

Supplementary Figure 29. (a) GITT curves of Li|LiTFSI-PEO-0.2AlF_3|FeF_3 cell measured at 60 ℃ with an intermittent period of 1h at 35 mA/g and then a relaxation time for 6 h during charge and discharge processes. (b) Voltage profiles of a single-step GITT at ~2.8 V during charging process. (c) Linear relationship and fitting between the transient potential (Es) and the square root of time.

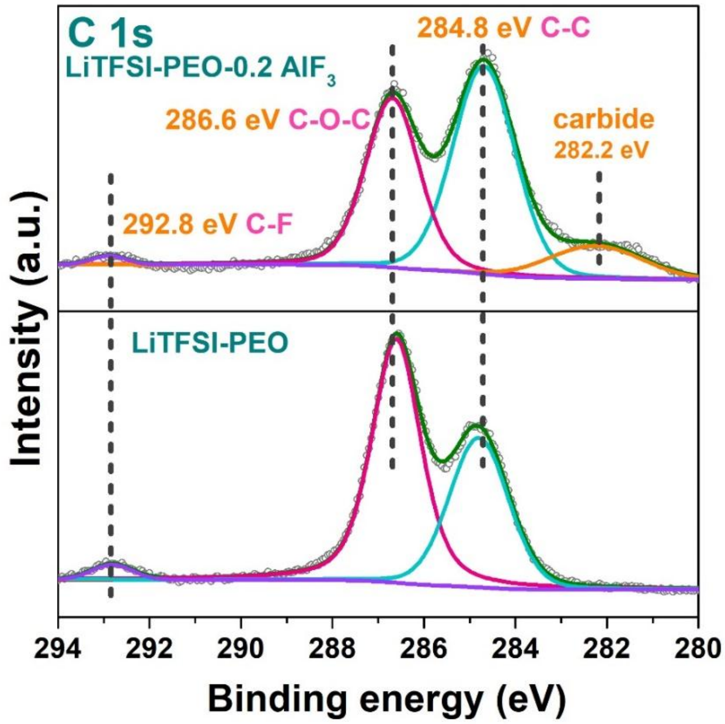

Supplementary Figure 30. Comparison of XPS spectra of C 1s signal for the cycled cathode (FeF_3+C+LiTFSI+PEO) at the terminal charging state after 10 cycles based on the LiTFSI-PEO-0.2AlF_3 and PEO-LiTFSI electrolytes. The corresponding cells for XPS measurement were cycled in a voltage range of 1.2-3.9 V at 358 mA/g at 60 ℃.

Supplementary Table 1. Simulation result of Supplementary Figure 7a according to the equivalent circuit in Supplementary Figure 7b.

Temperature (℃)	R_1		R_2		CPE_2-T		CPE_2-P	
	Value	Error (%)	Value	Error (%)	Value	Error (%)	Value	Error (%)
25	174.80	4.69	364.50	2.92	8.44E-09	9.29	0.81	2.62
30	128.80	5.53	226.30	4.95	1.28E-08	8.53	0.82	4.64
40	145.30	2.31	27.78	7.24	3.61E-11	7.92	0.95	5.06
50	90.42	2.03						
60	58.15	1.10E-10						
70	31.90	7.19						

Supplementary Table 2. Simulation result of the inset of Figure 4b according to the equivalent circuit in Supplementary Figure 9d.

Stage	R_b		R_{SEI}		R_{ct}	
	Value	Error (%)	Value	Error (%)	Value	Error (%)
Before polarization	35.54	0.67	222.40	0.98	23.33	7.02
After polarization	36.55	0.58	233.70	0.76	25.11	8.18

Stage	CPE_1-T		CPE_1-P		CPE_2-T		CPE_2-P	
	Value	Error (%)	Value	Error (%)	Value	Error (%)	Value	Error (%)
Before polarization	44.92E-06	4.96	0.78	0.64	1.10E-03	9.48	0.96	9.19
After polarization	5.02E-06	4.01	0.78	0.53	11.01E-03	8.41	0.97	6.19

Supplementary Table 3. Simulation results of the inset of Supplementary Figure 9a according to the equivalent circuit in Supplementary Figure 9d.

Stage	R_b		R_{SEI}		R_{ct}	
	Value	Error (%)	Value	Error (%)	Value	Error (%)
Before polarization	37.37	0.64	25.07	7.01	147.00	3.94
After polarization	35.86	0.69	50.37	8.61	240.00	3.81

Stage	CPE_1-T		CPE_1-P		CPE_2-T		CPE_2-P	
	Value	Error (%)	Value	Error (%)	Value	Error (%)	Value	Error (%)
Before polarization	3.59E-07	9.93	0.93	4.08	1.11E-06	8.68	0.92	1.67
After polarization	8.23E-07	9.49	0.92	2.65	1.13E-06	6.44	0.92	1.42

Supplementary Table 4. Simulation results of the inset of Supplementary Figure 9b according to the equivalent circuit in Supplementary Figure 9d.

.

Stage	R_b		R_{SEI}		R_{ct}	
	Value	Error (%)	Value	Error (%)	Value	Error (%)
Before polarization	366.80	0.56	29.91	5.26	208.50	1.58
After polarization	372.60	0.59	37.60	5.11	250.70	1.80

Stage	CPE_1-T		CPE_1-P		CPE_2-T		CPE_2-P	
	Value	Error (%)	Value	Error (%)	Value	Error (%)	Value	Error (%)
Before polarization	1.0E-06	8.52	0.95	2.55	2.15E-06	5.17	0.91	0.96
After polarization	9.50E-07	8.17	0.95	2.55	2.15E-06	5.95	0.91	1.13

Supplementary Table 5. Simulation results of the inset of Supplementary Figure 9c according to the equivalent circuit in Supplementary Figure 9d.

Stage	R_b		R_{SEI}		R_{ct}	
	Value	Error (%)	Value	Error (%)	Value	Error (%)
Before polarization	54.40	0.33	120.00	0.76	19.65	7.27
After polarization	50.47	0.27	95.98	0.73	47.74	7.00

Stage	CPE_1-T		CPE_1-P		CPE_2-T		CPE_2-P	
	Value	Error (%)	Value	Error (%)	Value	Error (%)	Value	Error (%)
Before polarization	4.05E-06	4.63	0.79	0.58	7.41E-04	8.18	0.80	8.08
After polarization	5.37E-06	3.90	0.80	0.50	8.18E-04	7.96	0.76	3.52

Supplementary Table 6. Simulation result of Figure 5b according to the equivalent circuit in the inset of Figure 5b.

Time (day)	R_b		R_{SEI}		R_{ct}	
	Value	Error (%)	Value	Error (%)	Value	Error (%)
1	16.28	1.17	11.90	7.42	14.33	9.26
5	14.15	9.79	11.98	7.27	12.99	6.53
10	10.43	0.49	5.52	7.11	19.26	4.15
15	9.33	1.44	7.21	7.42	17.33	8.99
20	15.75	0.64	9.41	6.65	16.14	6.38

Time (day)	CPE_1-T		CPE_1-P		CPE_2-T		CPE_2-P	
	Value	Error%	Value	Error%	Value	Error%	Value	Error%
1	2.35E-06	8.78	0.88	8.86	22.65E-05	8.25	0.79	4.83
5	5.49E-06	8.07	0.84	3.14	1.61E-05	8.09	0.87	2.08
10	2.20E-06	7.66	0.96	2.79	1.48E-05	3.51	0.80	0.97
15	1.98E-06	8.70	0.95	4.87	1.23E-05	8.23	0.85	1.76
20	9.30E-07	8.35	0.90	3.75	1.65E-05	5.41	0.80	2.53

Supplementary Table 7. Simulation result of Supplementary Figure 10 according to the equivalent circuit in the inset of Supplementary Figure 10.

Time	R_b		R_{SEI}		R_{ct}	
	Value	Error (%)	Value	Error (%)	Value	Error (%)
After 200 h	27.23	8.68	2.92	7.07	9.12	6.99

Time	CPE_1-T		CPE_1-P		CPE_2-T		CPE_2-P	
	Value	Error (%)	Value	Error (%)	Value	Error (%)	Value	Error (%)
After 200 h	1.32E-06	9.52	0.83	6.43	4.73E-05	7.23	0.84	3.37

Supplementary Table 8. Simulation results of Supplementary Figure 13a according to the equivalent circuit in the inset of Supplementary Figure 13a.

Time (day)	R_b		R_{SEI}		R_{ct}	
	Value	Error (%)	Value	Error (%)	Value	Error (%)
1	11.24	0.51	5.39	6.16	36.23	1.76
2	12.58	0.70	5.80	7.12	41.64	1.86
11	17.62	0.82	7.07	7.96	50.53	2.35
12	9.23	0.95	7.58	8.94	74.22	1.94
14	8.89	1.10	7.13	7.45	78.98	1.50

Time (day)	CPE_1-T		CPE_1-P		CPE_2-T		CPE_2-P	
	Value	Error (%)	Value	Error (%)	Value	Error (%)	Value	Error (%)
1	2.90E-06	7.84	0.99	2.48	3.80E-06	1.56	0.93	0.36
2	4.53E-06	8.69	0.95	3.24	3.46E-06	1.33	0.93	0.33
11	2.66E-06	8.16	0.99	2.81	2.55E-06	2.27	0.95	0.52
12	3.46E-06	8.41	0.96	3.23	2.53E-06	2.48	0.93	0.57
14	4.16E-06	7.26	0.95	2.53	2.58E-06	2.03	0.92	0.46

Supplementary Table 9. Simulation results of Supplementary Figure 24a according to the equivalent circuit in the inset of Supplementary Figure 24a.

Temperature (℃)	R_1		R_2		CPE_2-T		CPE_2-P	
	Value	Error (%)	Value	Error (%)	Value	Error (%)	Value	Error (%)
25	116.70	9.93	1451.00	2.87	6.65E-08	9.73	0.64	2.12
30	89.82	6.77	1156.00	2.92	7.09E-08	9.92	0.64	2.27
40	150.90	5.56	231.50	6.69	5.91E-09	9.90	0.88	6.13

Supplementary Table 10. Simulation results of Figure 7e according to the equivalent circuit in the inset of Figure 7e.

Stage (cycles)	R_b		R_{SEI}		R_{ct}	
	Value	Error (%)	Value	Error (%)	Value	Error (%)
Before	50.39	7.70	49.71	7.69	129.00	6.35
10	32.53	1.80	63.11	9.97	18.68	7.92
150	24.69	1.31	11.13	8.72	107.30	6.03
250	29.01	1.25	12.75	9.82	59.74	9.19
350	26.03	0.52	6.44	9.66	88.84	4.09

Stage	CPE_1-T		CPE_1-P		CPE_2-T		CPE_2-P	
	Value	Error (%)	Value	Error (%)	Value	Error (%)	Value	Error (%)
Before (cycles)	2.11E-06	6.23	0.81	4.15	1.20E-06	6.01	0.90	7.92
10	1.00E-05	9.21	0.73	2.49	1.47E-03	9.41	0.72	6.03
150	5.02E-07	7.81	0.79	7.23	1.65E-05	7.72	0.60	5.02
250	5.99E-08	8.90	0.97	5.32	7.85E-06	8.46	0.76	4.80
350	4.22E-08	6.12	0.97	3.48	1.98E-05	3.23	0.69	0.72

References

S1. Hu, J., Chen, K., Yao, Z. & Li, C. Unlocking solid-state conversion batteries reinforced by hierarchical microsphere stacked polymer electrolyte. *Sci. Bull.* **66**, 694-707 (2021).

S2. Wu, X. *et al.* Metal organic framework reinforced polymer electrolyte with high cation transference number to enable dendrite-free solid state Li metal conversion batteries. *J. Power Sources* **501**, 229946 (2021).

S3. Chen, H. *et al.* Stable seamless interfaces and rapid ionic conductivity of Ca–CeO_2/LiTFSI/PEO composite electrolyte for high-rate and high-voltage all-solid-state battery. *Adv. Energy Mater.* **10**, 2000049 (2020).

S4. Sheng, O. *et al.* $Mg_2B_2O_5$ nanowire enabled multifunctional solid-state electrolytes with high ionic conductivity, excellent mechanical properties, and flame-retardant performance. *Nano Lett.* **18**, 3104-3112 (2018).

S5. Wu, X. *et al.* Solid electrolytes reinforced by infinite coordination polymer nano-network for dendrite-free lithium metal batteries. *Energy Storage Mater.* **41**, 436-447 (2021).

S6. Li, S. *et al.* A superionic conductive, electrochemically stable dual-salt polymer electrolyte. *Joule* **2**, 1838-1856 (2018).

S7. Li, Y. *et al.* Hexagonal boron nitride induces anion trapping in a polyethylene oxide based solid polymer electrolyte for lithium dendrite inhibition. *J. Mater. Chem. A* **8**, 9579-9589 (2020).

S8. Wan, J. *et al.* Ultrathin, flexible, solid polymer composite electrolyte enabled with aligned nanoporous host for lithium batteries. *Nat. Nanotechnol.* **14**, 705-711 (2019).

S9. Wang, Z., Shen, L., Deng, S., Cui, P. & Yao, X. 10 μm-thick high-strength solid polymer electrolytes with excellent interface compatibility for flexible all-solid-state lithium-metal batteries. *Adv. Mater.* **33**, 2100353 (2021).

S10. Xu, H. *et al.* High-performance all-solid-state batteries enabled by salt bonding to perovskite in poly(ethylene oxide). *Proc. Natl. Acad. Sci.* **116**, 18815 (2019).
